# Modeling qualitative between-person heterogeneity in time series using latent class vector autoregressive models

**DOI:** 10.3758/s13428-025-02909-7

**Published:** 2025-12-26

**Authors:** Anja F. Ernst, Jonas M. B. Haslbeck

**Affiliations:** 1 Department Psychometrics & Statistics, University of Groningen, Grote Kruisstraat 2/1, 9712 TS Groningen, The Netherlands; 2 Psychological Methods Group, University of Amsterdam, Amsterdam, Netherlands; 3 Department of Clinical Psychological Science, Maastricht University, Maastricht, Netherlands

**Keywords:** Time-series, Temporal dynamics, Vector autoregressive modeling, Heterogeneity, Latent class modeling, Clustering

## Abstract

Time-series data have become ubiquitous in psychological research, allowing us to study detailed within-person dynamics and their heterogeneity across persons. Vector autoregressive (VAR) models have become a popular choice as a first approximation of these dynamics. The VAR model for each person and heterogeneity across persons can be jointly modeled using a hierarchical model that treats heterogeneity as a latent distribution. Currently, the most popular choice for this is the multilevel VAR model, which models heterogeneity across persons as *quantitative* variation through a multivariate Gaussian distribution. Here, we discuss an alternative, the latent class VAR model, which models heterogeneity as *qualitative* variation using a number of discrete clusters. While this model has been introduced before, it has not been readily accessible to researchers. Here we address this issue by providing an accessible introduction to latent class VAR models; a simulation evaluating how well this model can be estimated in situations resembling applied research; introducing a new R package *ClusterVAR*, which provides easy-to-use functions to estimate the model; and providing a fully reproducible tutorial on modeling emotion dynamics, which walks the reader through all steps of estimating, analyzing, and interpreting latent class VAR models.

## Introduction

Time-series data, which consist of a large number of repeated measures of a person, have become ubiquitous in psychological research (Hamaker et al., 2015; Kuppens & Verduyn, 2017; Myin-Germeys et al., 2009; Walls & Schafer, 2006), made possible by the widespread availability of mobile devices. These devices have greatly increased the feasibility of ecological momentary assessment (EMA) studies (Kuppens et al., 2022) and allow researchers to study a person’s subjective experiences in their natural environment with high temporal resolution (e.g., several measurements a day Hamaker & Wichers, 2017; Kuppens & Verduyn, 2017; Myin-Germeys & Kuppens, 2022). Such data promise a much more refined understanding of the dynamics of a person’s behavior and experiences, as well as how these dynamics differ between individuals. The widespread availability of such intensive longitudinal data from many persons raised the question of how to effectively model such data, resulting in numerous developments in statistical methodology to address these challenges (e.g., Bringmann et al., 2017; Ernst et al., 2024; Gates et al., 2023; McNeish & Hamaker, 2020; Oravecz et al., 2011; Ryan et al., 2018; Schuurman et al., 2016; Vogelsmeier et al., 2019).

Currently, the most popular model for capturing within-person dynamics in psychological time-series is the vector autoregressive (VAR) model, which models each variable at a given time point as a linear function of all variables (including itself) at the previous time point (Hamilton, 1994; Lütkepohl, 2005). This model captures the temporal dynamics between observed variables through lagged relationships: the conditional linear effect of a given variable at one time point on a given variable at the next time point. The effect of a variable on itself at a later time point, the autoregression, is often interpreted in terms of stability. Specifically in the context of emotion dynamics, this stability has been interpreted as the inertia or persistence of experiences from one moment to the next and in the case of negatively valenced emotions has been linked to several mental health outcomes (Koval et al., 2012; Kuppens & Verduyn, 2017). The cross-lagged regressions between two variables, on the other hand, are considered candidates for causal effects between the modeled variables (Hamaker et al., 2018; Masten & Cicchetti, 2010).

Beyond studying within-person dynamics that are present in time-series data, researchers aim to capture systematic heterogeneity across persons in these dynamics (Beck & Jackson, 2020; Brose et al., 2015; Hoekstra et al., 2023; Van der Krieke et al., 2017; Wright et al., 2015). One way to explore heterogeneity across persons is to estimate person-specific models and account for the heterogeneity between these models in a second step. However, a joint model is generally better because otherwise the model for heterogeneity does not incorporate the uncertainty about the estimates from the first step. One negative consequence is that we might end up modeling heterogeneity that is actually due to sampling variability. In addition, in many situations, time series of individual persons are too short to support estimating VAR models with more than a few variables (Bulteel et al., 2018; Dablander et al., 2020; Lafit et al., 2022; Liu, 2017; Mansueto et al., 2022). Many researchers therefore adopt a multi-person approach where the between-person heterogeneity is modeled alongside the dynamics in a single longitudinal model. This between-person heterogeneity can be captured using continuous or discrete latent variables.

Continuous latent variables, such as random coefficients, can model between-person heterogeneity that is dimensional in nature and can therefore be well approximated with a continuous latent distribution around an average parameter (De Leeuw & Meijer, 2008). Commonly, this continuous distribution is assumed to be multivariate Gaussian for both practical and theoretical reasons (e.g., McElreath, 2018). Methods that capture between-person heterogeneity of VAR models through continuous latent variables include multilevel VAR models (Driver & Voelkle, 2018; Epskamp et al., 2018; Bringmann et al., 2013; Li et al., 2022; McNeish & Hamaker, 2020; Rovine & Walls, 2006) or dynamic structural equation modeling (DSEM, Hamaker et al., 2018). The nature of these methods is that person-specific estimates are shrunk towards the average parameter estimate, proportional to how many time points are available for the person at hand and how far away the person-specific estimate is from the average parameter estimate (De Leeuw & Meijer, 2008). This approach generally leads to better estimates if the heterogeneity in VAR parameters is well approximated by a multivariate Gaussian distribution (e.g., McElreath, 2018). However, if the heterogeneity is structured in a number of subgroups, this structure will be masked by this approach because all estimates are shrunk towards the same average parameter (Ernst et al., 2024).

Another approach to modeling heterogeneity between people is to use categorical latent variables. Here, heterogeneity is captured by categorizing persons into homogeneous subgroups that can differ qualitatively from each other. These latent categorical variables can be seen as subgroups and are identified with either a clustering model or algorithm (Lubke & Muthén, 2005). Here we refer to these subgroups as *clusters*, but depending on the literature they are also referred to as mixture components or latent classes (McLachlan & Basford, 1988; Vermunt & Magidson, 2003). Clusters are a powerful approach to account for heterogeneity, because they can accommodate qualitatively different time-series models across persons, which are often observed in practice (Brose et al., 2015; Hamaker et al., 2016) and are theoretically expected across many domains of psychological research. For example, emotional dynamics are assumed to differ qualitatively across persons (Houben et al., 2015) due to differences in how situations are perceived (Barrett, 2018) or how emotion regulation strategies are employed (Hay & Diehl, 2011). For a more detailed discussion on the distinction between continuous and categorical latent variable models, see Appendix A.

Latent class VAR (LCVAR) models are perhaps the most direct way to implement the idea of (qualitative) heterogeneity across VAR models of different persons using a categorical latent variable (Ernst et al., 2020). These models classify persons into different clusters in such a way that the VAR models within a cluster are more similar to each other than to those in other clusters. In LCVAR models, all persons remain in the same cluster throughout.^1^ Classifications are done in an exploratory fashion, meaning they are not known a priori. Related methods include group iterative multiple model estimation (GIMME, Gates & Molenaar, 2012; Lane & Gates, 2017), VAR clustering through alternating least squares (Bulteel et al., 2016), and sub-grouping in the multi-VAR framework (Crawford et al., 2024). Here we focus on LCVAR models for the following reasons: First, the estimation of the model and cluster memberships is straightforward in LCVAR by assigning persons into clusters in a way that maximizes the likelihood function, and hence does not depend on tuning parameters or arbitrary cut-off values (in contrast to, for instance, GIMME, Gates & Molenaar, 2012; Lane & Gates, 2017). Second, LCVAR offers the advantage that it combines the estimation of VAR model parameters and cluster memberships so that they can mutually inform one another. This is in contrast to other VAR-clustering methods that use a two-step estimation, which first estimate person-specific VAR models and then cluster them in a separate step without allowing the clustering to influence the way the VAR parameters are estimated (e.g., Ernst et al., 2021; Gates & Molenaar, 2012; Lane & Gates, 2017). Finally, LCVAR identifies clusters of persons based on similarities in the *values* of VAR model parameters, while other VAR-clustering methods only consider whether or not parameters are estimated to be equal to zero or not when estimating clusters (e.g., Crawford et al., 2024; Gates & Molenaar, 2012; Hoekstra et al., 2023).

Despite LCVAR being a powerful model to estimating VAR models that accommodate qualitative heterogeneity across persons, it has not yet been widely adopted in psychological research. We think this is due to four reasons: First, there is no accessible introduction to LCVAR models geared towards EMA researchers. Second, there is no systematic evaluation of how well LCVAR models can be recovered from time series that mirror typical EMA designs. Third, there is no easy-to-use R package that implements the method. Finally, there is no accessible tutorial guiding researchers through the steps of fitting LCVAR models, performing model selection, evaluating models, and interpreting them correctly. In this paper, we remove these obstacles.

We begin by providing an accessible introduction to modeling qualitative heterogeneity in VAR models with the LCVAR model (Ernst et al., 2020). We then present an extensive simulation study showing which types of heterogeneity can be recovered and how the model performs across many empirically relevant situations. These results help researchers interpret their empirical findings and design new studies capable of detecting qualitative heterogeneity in the dynamics captured by the LCVAR model. Finally, we provide a fully reproducible tutorial showcasing a new R package, *ClusterVAR*, and demonstrating how to use it to estimate LCVAR models, perform model selection, and interpret its parameter estimates. We conclude by discussing how to best model heterogeneity across persons in temporal dynamics and highlighting desirable methodological improvements.

## Latent class vector autoregressive (LCVAR) models

In this section, we describe the LCVAR model, in which each person is assumed to belong to exactly one of *K* clusters. We model each person’s time series using a VAR model parameterized by means and a time-lagged effects matrix, with autoregression on the diagonal and cross-lagged regressions on the off-diagonal. In the LCVAR model, a person’s VAR model parameters depend on their cluster membership. The LCVAR model assumes a fixed number of clusters *K*, each described by a fixed VAR model.

Thus, the LCVAR model assumes that the underlying VAR model for all persons in a given cluster is exactly the same. Naturally, this assumption is unlikely to hold exactly in empirical data. We will revisit this issue in the simulation study, where we examine how well the LCVAR model can be recovered from data when parameter variation *does* exist between persons within the same cluster. We will also further discuss this issue in the Tutorial and Discussion sections.

### LCVAR model specification

As proposed in Ernst et al. (2020), the LCVAR model for an observed multivariate time-series $\boldsymbol{y}_{i}$ of person *i* ($i = 1, 2, \ldots , N$) with *p* lags can be specified as follows

1 $$\begin{aligned} \text {Level 1 (time points)}: \boldsymbol{y}_{i\; t}= &  \boldsymbol{w}_{i\; t}\, + \boldsymbol{\mu }_i + \boldsymbol{\beta }_{i} \boldsymbol{x}_{i\; t}, \end{aligned}$$

2 $$\begin{aligned} \boldsymbol{w}_{i\;t}= &  \left( \sum _{a = 1}^{p} \boldsymbol{\Phi }_{i\; a} \boldsymbol{ w}_{i,\; t\,-\,a}\,\right) + \, \boldsymbol{u}_{i\; t}\qquad \boldsymbol{u}_{i\; t} \sim N(\boldsymbol{ 0},\, \boldsymbol{\Sigma }_{i}) \end{aligned}$$

where $\boldsymbol{y}_{i\; t}$ is a vector of *m* observed outcome variables at time point $t \in \{1,2,\ldots ,T_{i} \}$). $\boldsymbol{\mu }_{i}$ is the $m \times 1$ vector that contains the (moderated) within-person mean for each of these variables (Hamaker et al., 2018, 2015). Here, the subscript *i* indicates that this mean is person-specific, which in the LCVAR model means that it is cluster-specific.

The matrix $\boldsymbol{\beta }_{i}$ is a $m \times q$ matrix that indicates immediate influences of *q* exogenous variables (i.e., covariates), $\boldsymbol{x}_{i\; t}$, on the means, $\boldsymbol{\mu }_{i}$, (for an interpretation of these influences, see Ernst et al., 2024). These exogenous variables thus moderate the means. For instance, the means could be moderated by the time-of-day of the measurement. By including a dummy for morning measurements in $\boldsymbol{x}_{i\; t}$, this model can estimate a different mean for morning measurements than for non-morning measurements (Bringmann et al., 2024; Ernst et al., 2025). $\boldsymbol{w}_{i\; t}$ is a time-series of *m* endogenous variables (i.e., outcome variables) which excludes the (moderated) mean, this time-series is a within-person process (Hamaker et al., 2018, 2015).

$\boldsymbol{\Phi }_{i\; a}$ is the $m \times m$ time-lagged effects matrix containing autoregressions (on the diagonal) and cross-lagged regressions (on the off-diagonal) of the outcome variables at time lag *a*. Usually, all $\boldsymbol{\Phi }_{i\; a}$ for all *p* time-lags are collected into a single matrix, $\boldsymbol{\Phi }_{i}$, for simplicity. $\boldsymbol{u}_{i\; t}$ are innovations that are distributed with $m \times m$ covariance $\boldsymbol{\Sigma }_{i}$. Usually, particular interest lies on $\boldsymbol{\Phi }_{i}$, as it constitutes the dynamics with which different variables develop and influence one another from one time point to the next, and $\boldsymbol{\mu }_{i}$, because it constitutes the means of the *m* outcome variables across all time points. At least this is the interpretation of $\boldsymbol{\mu }_{i}$ as long as the means are unmoderated, that is, as long as no exogenous variables, $\boldsymbol{x}_{i\; t}$, are included. If any exogenous variables are included, $\boldsymbol{\mu }_{i}$ constitute the means across all time points when $\boldsymbol{x}_{i\; t} = \boldsymbol{0}$.

In a latent class model, every person, *i*, is assumed to belong to exactly one of *K* ($k = 1, 2, \ldots , K$) clusters (i.e., latent classes) (McLachlan & Basford, 1988; McLachlan & Peel, 2004; Vermunt & Magidson, 2003). In the LCVAR model, the parameters of the VAR model of a specific person depend on which cluster the person belongs to in the following way

3 $$\begin{aligned} \text {Level 2 (persons)}:&\boldsymbol{\mu }_{i} = \sum _{k = 1}^{K} z_{i\; k} \boldsymbol{\mu }_{k} \end{aligned}$$

4 $$\begin{aligned}&\boldsymbol{\beta }_{i} = \sum _{k = 1}^{K} z_{i\; k} \boldsymbol{\beta }_{k} \end{aligned}$$

5 $$\begin{aligned}&\boldsymbol{\Phi }_{i\; a} = \sum _{k = 1}^{K} z_{i\; k} \boldsymbol{\Phi }_{k \; a} \end{aligned}$$

6 $$\begin{aligned}&\boldsymbol{\Sigma }_{i} = \sum _{k = 1}^{K} z_{i\; k} \boldsymbol{\Sigma }_{k}. \end{aligned}$$

where the person’s VAR parameters are equal to the cluster-specific VAR parameters of the cluster the person belongs to. Cluster-specific parameters represent the average parameters across all persons who belong to a given cluster. In the equation above, $z_{i\; k}$ serves as an indicator variable of cluster membership, where $z_{i\; k} = 1$ if person *i* is a member of cluster *k* and 0 otherwise.

LCVAR makes the following additional assumptions. Innovations $\boldsymbol{u}_{i\; t}$ are assumed to be normally distributed with a mean of zero and covariance matrix $\boldsymbol{\Sigma }_{k}$ (Lütkepohl, 2005). Further, innovations are assumed to be serially independent of one another and independent of all predictors that appear with them in the regression equation (i.e., $\boldsymbol{u}_{i\; t}$ is independent of $\boldsymbol{x}_{i\; t}$ and of the previous values of the outcome variables, $\boldsymbol{w}_{i,\; t\,-\,a}$; Wooldridge, 2009, p. 351). Furthermore, as in standard VAR models, the time series is assumed to be a weakly stationary Gaussian process, meaning that the variances and autocovariances of the outcome variables remain stable over time (Ryan et al., 2024; Hamilton, 1994). Lastly, as in all discrete-time VAR models, all time points in the time series are assumed to be equidistant.

### LCVAR model estimation

The true cluster membership of person *i* to belong to cluster *k*, $z_{i\; k}$, is unknown, but the cluster membership probability, $\hat{\pi }_{i\;k}$, can be estimated with Bayes’ rule in the following way, given the person’s observed time series, $\boldsymbol{Y}_{i}$, estimates for the cluster-specific VAR parameters for each cluster, collected in $\hat{\boldsymbol{\Theta }}_k$, and the estimated proportion of persons contained in cluster *k*, $\hat{\tau }_{k}$, (i.e., the estimated prior probabilities of cluster membership):

7 $$\begin{aligned} \hat{\pi }_{i\;k} = \frac{ \hat{\tau }_{k} \prod _{t=1}^{T_{i\; k}} \Delta (\boldsymbol{y}_{i\, t};\, \hat{\boldsymbol{\Theta }}_k) }{\sum _{k' = 1}^{K} \hat{\tau }_{k'}\prod _{t=1}^{T_{i\; k'}}\Delta (\boldsymbol{y}_{i\, t};\, \hat{\boldsymbol{\Theta }}_{k'})}. \end{aligned}$$

In our notation, we use a hat on top of $\hat{\tau }_{k}$ to indicate that this is the estimate of a parameter rather than the true parameter. $\Delta (\boldsymbol{y}_{i\, t};\, \hat{\boldsymbol{\Theta }}_k)$ denotes the conditional density function of observing $\boldsymbol{y}_{i\, t}$, given the *p* previous observations, $\boldsymbol{y}_{i\, t-1}$ to $\boldsymbol{y}_{i\, t-p}$, and the estimates for cluster-specific parameters of cluster *k*, $\hat{\boldsymbol{\Theta }}_k$ (Michael & Melnykov, 2016). In words, Eq. 7 estimates the probabilistic cluster membership of person *i* to belong to cluster *k* by weighting the likelihood of observing the time-series of person *i* if it had been generated by the cluster-specific estimates of cluster *k*, $\hat{\boldsymbol{\Theta }}_k$, against the likelihood of observing the same time-series if it had been generated by the cluster-specific estimates of any of the other clusters. Equation 7 additionally takes into account the relative number of persons assigned to cluster *k* compared to other clusters by using the estimated prior probabilities, $\hat{\tau }$.

Equation 7 shows that we can calculate the probabilistic cluster memberships given an estimate of the cluster-specific parameters and prior probabilities. However, to estimate the cluster-specific parameters and prior probabilities based on the observed data, we require an estimate for the probabilistic cluster memberships. One way to resolve this issue is to estimate all these parameters using the expectation-maximization (EM) algorithm (Dempster et al., 1977), which is an iterative algorithm that can combine the estimation of cluster-specific parameters, such as $\boldsymbol{\Phi }_{k}$ and $\boldsymbol{\mu }_{k}$, prior probabilities, and probabilistic cluster memberships into iterative estimation steps. Specifically, the algorithm starts with an initialization (i.e., a first guess) of cluster memberships^2^ and then uses these memberships to estimate the cluster-specific parameters and prior probabilities. In a subsequent step, the cluster membership probabilities of each person are updated in line with Eq. 7 based on the estimates of the previous step. These two steps are repeated iteratively until convergence, which is determined when the log-likelihood of the LCVAR model no longer improves beyond a predefined convergence criterion. Multiple initializations are typically used for the EM algorithm to avoid ending in a local maxima. An EM algorithm for estimating the LCVAR model was proposed by Ernst et al. (2020), we refer to this paper for details on its implementation.

#### Lag orders and the effective number of time points

In the notation above, we considered the scenario where the lag order *p* is equal across all clusters. However, LCVAR models can also be estimated with different lag orders for each cluster (Ernst et al., 2020). In such cases, the lag order of cluster *k* is denoted by $p_k$. When considering different combinations of lag orders across the different clusters (e.g., two clusters with one lag and one cluster with two lags), the order of the combination does not matter because the ordering of the clusters is arbitrary.

In any VAR model, including LCVAR, the lag order determines the effective number of time points available to estimate the model. The effective number of time points of person *i*, $T_{i\, k}$, contains only those time points that can be predicted based on the available data $\textemdash$ specifically, those for which all $p_k$ previous time points have observed values ($p_k$ is the lag order in cluster *k* to which person *i* belongs). Therefore, the first $p_k$ time points of a time-series can never be predicted and if an observation at a given time point is missing, the following $p_k$ time points cannot be predicted either. In the simplest case, for example, for a VAR model with lag 1, the first time point in the time-series must be excluded, since there clearly is no previous time point to use to predict the first time point. Similarly, in a model with lag 3, the first three time points must be excluded since they cannot be predicted. The number of excluded time points increases markedly in EMA designs, where time-series effectively restart on every day and/or when there are missing observations within a day.

### Selecting the lag order combination and the number of clusters

So far, we have discussed the LCVAR model and its estimation assuming we already know the lag order combination across clusters (e.g., two clusters with 1 lag and one cluster with 2 lags) and the number of clusters, K. In practice, these are not known a priori. To address this, we discuss two information criteria for model selection $\textemdash$   Hannan-Quinn criterion (HQ) and Schwarz criterion (SC) $\textemdash$   for selecting a combination of lag orders for all clusters, and two information criteria $\textemdash$   Bayesian information criterion (BIC) and Integrated Complete-data Likelihood (ICL) $\textemdash$   for selecting the number of clusters. Crucially, no existing information criterion can simultaneously select both the combination of lag order and the number of clusters. Below we provide a technical explanation why such simultaneous selection is not possible with these information criteria. In the Discussion section, we provide guidance for empirical researchers on selecting a final model regarding the lag order combination and the number of clusters. In our simulation study, we evaluate the ability of these different information criteria to recover the underlying combination of lag orders or the number of clusters across various empirically relevant scenarios.

#### Information criteria to select the lag order combination

The optimal combination of lag orders for a given number of clusters can be determined using the time-series information criteria HQ and SC, which balance forecasting precision against the number of effective time points and the number of time-series parameters (Akaike, 1969, 1971, 1974; Quinn, 1980). Because these information criteria were originally developed for selecting the lag order of a single time-series, we extend them to the case of multiple clusters to assess model fit across all clusters. In these extension to the cluster case, the forecasting precision in each cluster is balanced with the estimated proportion of persons in the cluster, $\hat{\tau }_k$, and the number of effective time points that are contained in the cluster. This number of effective time points for a given cluster *k* is determined by weighing the number of effective time points for each person, $T_{i\, k}$, by the posterior probability that the person belongs to cluster *k*, $\hat{\pi }_{i\, k}$. The formulas for the extension of the HQ and SC for the cluster case are shown in Equations 8 and 9 in Appendix B.

The time-series information criteria identify the optimal model across all lag order combinations given a fixed number of clusters, *K*, selecting thus the model with the ideal combination of lag orders for all clusters (indicated as the model with the *lowest* information criterion across all candidate models). Crucially, however, because these time-series information criteria are weighted across clusters, they do not consider the total number of parameters estimated in an LCVAR model but only consider the number of time-series parameters within the clusters. Thus, these information criteria cannot be used to determine the number of clusters, because the number of parameters they consider does not increase as the number of clusters increases. These time-series information criteria can thus only compare LCVAR models with the same number of clusters.

#### Information criteria to select the number of clusters

To determine the best-fitting number of cluster we consider the information criteria BIC (Schwarz, 1978) and ICL (Biernacki et al., 2000). Note that the selection of the optimal number of clusters interacts with the selection of the number of lags. Here we consider the problem of selecting the optimal number of clusters while keeping the number of lags fixed and equal across all clusters. These global information criteria weigh the overall model fit against the total number of parameters in the model. The definitions of the BIC and ICL are given in Equations 10 and 11 in Appendix B. In general, the BIC weighs the model fit, quantified by the log likelihood, against the number of parameters and the number of effective time points. The ICL is an extension of the BIC in that it adjusts the BIC by how certain the classification is for each person (i.e., what is the posterior probability of a person for the cluster to which that person is assigned to based on modal posterior probability). Thus, the difference between BIC and ICL is that ICL additionally favors well-separated clusters (Biernacki et al., 2000). In many clustering models, such as mixture models and latent class models, the BIC is a widely used information criterion that typically performs well in selecting the optimal number of clusters (Nylund et al., 2007).

Above, we pointed out that the BIC and ICL cannot be used to select the combination of lag order and the number of clusters simultaneously. The reason is as follows. As explained in Section “Lag orders and the effective number of time points”, the number of effective time points, $T_{i\, k}$, depends on the lag order of cluster *k*. This dependence is particularly pronounced in empirical datasets with many missing observations or when the first observation of a day cannot be predicted from previous observations, leading to significant discrepancies in effective time points between models with different lag orders. For example, in a lag 1 model, if an observation is missing, the observation at the next time point cannot be predicted. In contrast, in a lag 2 model, if an observation is missing, the observations at the next two time points cannot be predicted. Since the likelihood of a model depends on the effective number of time points used for estimation, the likelihoods of models with different effective time points cannot be compared. As a result, models with different lag order combinations cannot be compared because their likelihoods are based on different numbers of effective time points. Thus, because BIC and ICL use likelihood values to compare models in terms of fit, they cannot compare models with different lag order combinations but can only compare models with the same lag order for all clusters.

## Simulation study: Evaluating recovery of LCVAR models

The goal of our simulation study is to evaluate how well LCVAR models can be estimated in situations that resemble typical EMA designs. Specifically, we investigate how accurately the number of clusters and the lag order combination can be recovered using the information criteria discussed above, and we assess how well the parameters of the LCVAR model can be estimated. We also consider situations in which the LCVAR model is misspecified by generating individual VAR parameters with heterogeneity within clusters. We included this type of misspecification because, to some extent, it occurs in virtually all empirical datasets.

### Simulation method

Here we outline which factors we varied or kept constant in our simulation. We then provide a brief overview of how LCVAR models were estimated and how performance was evaluated. All materials used in this simulation are available on OSF.^3^ The supplementary materials include the R scripts used for the simulation study and allow the reader to reproduce all results in the paper.

#### Simulation conditions

We varied the following six factors in a fully crossed design: (1) the number of clusters, (2) the number of lags, (3) the total number of persons ($N_{subj}$), (4) the number of time points per person ($N_{T}$), (5) whether between-cluster differences were contained only in lagged effects $\boldsymbol{\Phi }_k$ or only in means $\boldsymbol{\mu }_k$, and (6) the within-cluster variance (indicating overlap between clusters). We selected the levels of these six factors to reflect applied research in the following way:

1. *The number of clusters*, *K*, was equal to 1, 2, or 3. We chose these numbers because, we wanted to include in our simulation the scenario where there is no clustering of individuals (i.e., $K=1$). Because it is good practice to consider all smaller numbers of clusters when evaluating a certain number of clusters, comparisons between one, two, and three clusters are almost always included when comparing the fit of models with different numbers of clusters.
2. *The number of lags*, *p*, was equal to 1 or 2 for all clusters. We chose these numbers because, lag 1 models are usually of biggest interest, and in most applied research contexts there is not enough data to estimate a model with more than 2 lags. The number of lags was equal across all clusters. Thus in the $p=1$ conditions, all clusters had an underlying lag 1 time-series while in the $p=2$ conditions, all clusters had an underlying lag 2 time-series.
3. *The total number of persons*, $N_{subj}$, was equal to 50 or 200. We picked these values because the sample sizes for many EMA studies lie within this range (Ryan et al., 2024; Wrzus & Neubauer, 2023).
4. *The number of time points for each person*, $N_T$, was equal to either 50, 100, or 200 for all persons, to reflect a number of time points which are frequently employed in applied EMA research (Ryan et al., 2024; Wrzus & Neubauer, 2023).

The overlap between two clusters depends on both the distances between the cluster-specific parameters and the variation around those cluster-specific parameters within a cluster. We illustrate this relationship in Fig. 10 in Appendix C, where any degree of overlap between the clusters can be achieved by manipulating either the between-cluster distances of cluster-specific parameters, or by manipulating the within-cluster variance around these parameters. Thus, the overlap between two clusters can be expressed as the between-cluster distance relative to the within-cluster variance. In our simulation we choose to manipulate the overlap between clusters by keeping distances between cluster-specific parameters fixed and varying the within-cluster variance. We also varied an additional factor: whether the fixed between-cluster distances were present in either the lagged effects matrix ($\boldsymbol{\Phi }_k$) or in the means ($\boldsymbol{\mu }_k$). Below we first describe how this factor was implemented in our simulation, followed by a description of how we varied the within-cluster variance.

5. We varied whether *between-cluster differences* were present only in the lagged effects matrix ($\boldsymbol{\Phi }_k$) or only in the means ($\boldsymbol{\mu }_k$). In empirical data sets, between-cluster differences are likely to be spread over both $\boldsymbol{\Phi }_k$ and $\boldsymbol{\mu }_k$ to varying degrees. However, if researchers within-person center the data before analysis, all between-person differences (and thus all between-cluster differences) in $\boldsymbol{\mu }_k$ are removed and only differences in $\boldsymbol{\Phi }_k$ remain. This approach is ideal for researchers seeking to identify clusters that are homogeneous purely in terms of $\boldsymbol{\Phi }_k$. In our simulation study, we employed a design where between-cluster differences were present in only one type of parameter at a time (i.e., either in $\boldsymbol{\Phi }_k$ or in $\boldsymbol{\mu }_k$) to provide researchers with an upper bound performance (i.e., if all between-cluster differences are present in the type of parameter that leads to the best performance) and a lower bound performance (i.e., if all between-cluster differences are present in the type of parameter that leads to the worse performance). Depending on the spread of the between-person differences between these two types of parameters for a given data set, researchers can expect a performance that is falling between these bounds. We provide the details on how we implemented between-cluster differences in our simulation in Appendix C.
6. We varied the overlap between clusters by keeping fixed the between-cluster distances and varying the *within-cluster variance*. We introduced within-cluster variance in the following way: For each individual, all parameters contained in $\boldsymbol{\mu }_i$ and all lag 1 parameters contained in $\boldsymbol{\Phi }_i$ were drawn from a multivariate normal distribution with the mean equal to the respective cluster-specific parameters, $\boldsymbol{\mu }_{k}$ and $\boldsymbol{\Phi }_{k}$, of the cluster individual *i* belongs to (*k*), with a given variance (representing the within-cluster variance) and zero covariance. In the lag 2 conditions, the lag 2 parameters in $\boldsymbol{\Phi }_i$ contain no within-cluster variance and also no between-cluster differences. We made this choice in order to keep the total amount of within-cluster variance and between-cluster differences equal between the lag 1 and the lag 2 conditions. The only difference between these conditions was the amount of parameters requiring estimation.^4^ While between-cluster distances were contained in only $\boldsymbol{\Phi }_k$ or only in $\boldsymbol{\mu }_k$, within-cluster variance was always present in both, the parameters of $\boldsymbol{\boldsymbol{\Phi }}_k$ (always only the lag 1 parameters) and of $\boldsymbol{\mu }_k$. The within-cluster standard deviation of all parameters contained in $\boldsymbol{\mu }_i$ and all lag 1 parameters contained in $\boldsymbol{\Phi }_i$ varied at four levels: $\text {SD} \in$ $\{0, 0.03, 0.0424, 0.085 \}$.^5^ We selected these four within-cluster standard deviation values based a small pilot simulation study, which indicated that they would yield a wide range of model performance outcomes (from good to poor).

We are particularly interested in how performance is impacted by within-cluster variance (i.e., differences in individual parameters among persons in the same cluster) because the LCVAR model does not account for within-cluster variance, while such variance is likely to be present in almost any empirical data.

Across all ($3 \times 2 \times 2 \times 3 \times 2 \times 4 = 288$ in total) resulting simulation conditions, the following factors were kept fixed. The number of variables was set to $m = 6$. The innovation covariances, $\boldsymbol{\Sigma }_{k}$, contained 1 on the diagonal and 0.5 on the off-diagonal, similar to previous simulation studies (Lafit et al., 2022; Liu, 2017). The distribution of mixing proportions was equal across clusters: $\frac{1}{K}$.^6^

The number of replications was set to 100. In each replication, we estimated LCVAR models with one to four clusters. We chose this cluster range to ensure that the true number of clusters was always included, along with a model with more clusters than the true number. This allowed us to assess whether the information criteria selected the true number of clusters or if they exhibited a tendency to over-estimate or under-estimate it. For each number of clusters, all possible combinations of lag 1 and lag 2 across all clusters were estimated. After estimating all models, the time-series information criteria, SC and HQ, each selected the model with the best-fitting combination of lag 1 and lag 2, given the true underlying number of clusters in that replication condition.^7^ Conversely, the global information criteria, BIC and ICL, selected the model with the best-fitting number of clusters, given the true underlying lag order combination. For instance, if the true underlying lag order combination was lag 1 in all clusters, the global information criteria selected one model from four candidate models with different number of clusters: (A) A one cluster model with 1 lag in that cluster, (B) a two cluster model with 1 lag in both clusters, (C) a three cluster model with 1 lag in all three clusters, or (D) a four cluster model with 1 lag in all four clusters.

#### Estimating LCVAR models

We estimated the models with the LCVAR() function from the *ClusterVAR* R package (Ernst & Haslbeck, 2024, version 0.0.5).^8^ The number of random starts was set to 50, in addition to a rational start and a start based on a previous solution. The maximum number of EM-iterations was set to 100, and the convergence criterion was set to 1e-06.

#### Performance measures

To evaluate the estimation performance, we considered (1) model recovery $\textemdash$  how well the true model was recovered by the information criteria, and (2) estimation recovery $\textemdash$  how well the parameters of the model were recovered when estimating a model with the correct lag order combination and number of clusters. The following performance measures were used: The first performance measure assessed model recovery, while the last two assessed estimation recovery.

1. Model recovery accuracy was quantified as the proportion of correctly identified models: how frequently the true underlying lag order combination was selected by HQ and SC, or how frequently the true underlying number of clusters was selected by BIC and ICL.
2. Parameter recovery was quantified as the mean absolute deviation between estimated and true parameters for $\boldsymbol{\mu }_k$ and for $\boldsymbol{\Phi }_k$. For this we used the estimated parameters from a model with the correct lag order combination and number of clusters.
3. Cluster membership recovery was quantified using the adjusted Rand index (ARI) (Hubert & Arabie, 1985), which compared the modal estimated cluster membership probabilities (i.e., each individual was assigned to the cluster for which they had the highest estimated membership probability) with the true cluster membership from the data generation process. For this we used the estimated cluster membership probabilities from models with the correct lag order combination and number of clusters. The ARI is a measure of agreement between classifications, ranging from $-1$ to 1, where a value of 1 indicates perfect agreement between classifications and a value of 0 indicates that the agreement between classifications is what would be expected by chance. According to Steinley (2004), ARI values above 0.9 indicate excellent recovery, values above 0.8 indicate good recovery, and values below 0.65 indicate poor recovery.

### Simulation results

We first report how accurately the true model was recovered by the information criteria before discussing the recovery of model parameters. We then provide recommendations for researchers based on our simulation findings.

#### Model recovery

**Fig. 1:**
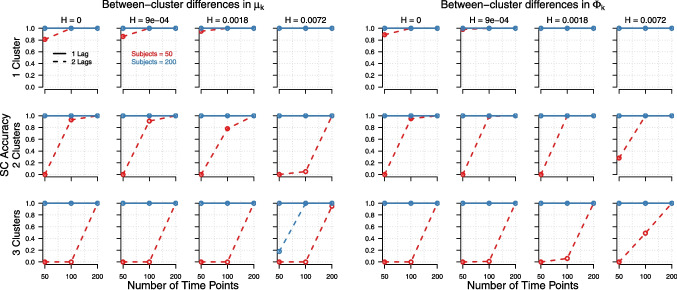
Accuracy of SC (i.e., proportion of times SC selected the correct lag order combination), as a function of the number of true clusters (*rows*), the within-cluster variance (*columns*), the number of time points (*x-axis*), the number of subjects (*red vs. blue*), and the number of lags (1 lag = *solid*, 2 lags = *dashed*). *Left panel*: When between-cluster differences were present in the means, $\boldsymbol{\mu }_k$. *Right panel*: The corresponding results when between-cluster differences were present in lagged effects matrix, $\boldsymbol{\Phi }_k$

**Fig. 2:**
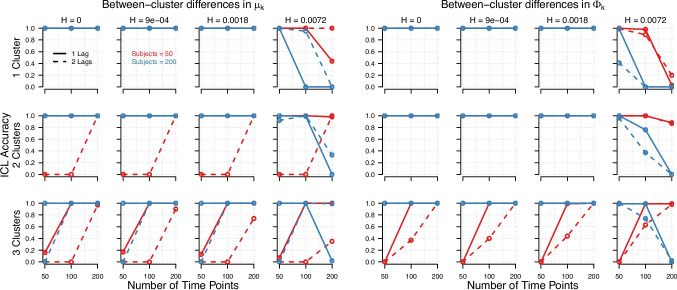
Accuracy of ICL (i.e., proportion of times ICL selected the correct number of clusters), as a function of the number of true clusters (*rows*), the within-cluster variance (*columns*), the number of time points (*x-axis*), the number of subjects (*red vs. blue*), and the number of lags (1 lag = *solid*, 2 lags = *dashed*). *Left panel*: When between-cluster differences were present in the means, $\boldsymbol{\mu }_k$. *Right panel*: The corresponding results when between-cluster differences were present in lagged effects matrix, $\boldsymbol{\Phi }_k$

First, we discuss overall effects on model recovery that are common to both lag selection and cluster selection. We then examine the recovery of lag order combinations before taking a closer look at recovery of the number of clusters. Overall, the accuracy of recovering the data-generating model was higher when between-cluster differences were in $\boldsymbol{\Phi }_k$ rather than in $\boldsymbol{\mu }_k$ (see Figs. 1 and 2). Model recovery was more accurate when the true model had 1 lag rather than 2 lags, and it declined as the number of clusters increased. Across conditions, the accuracy of model recovery was higher in conditions with more persons and more time points, but it decreased with higher within-cluster variance, especially for the recovery of the number of clusters. The main effects (i.e., averaged over all other simulation factors) for each of the six simulation factors on model recovery are summarized in Table 2 in Appendix D.

Here, we consider the selection of the combination of lag orders. SC performed poorly when the number of persons or time points was low. As shown in Fig. 1, SC required a high number of persons and/or time points to achieve satisfactory model recovery when the true model was a 2 lag model in all clusters. HQ recovered lag order combinations satisfactory across all conditions, except when the within-cluster variance was high and the number of persons and time points was also high (see Fig. 11 in Appendix D). HQ often outperformed SC in recovering the lag order combination, as seen when comparing Figs. 1 and 11 in Appendix D. SC performed better when the true number of lags was 1, whereas HQ performed better when the true number of lags was 2.

The accuracy of ICL in selecting the correct number of clusters, as shown in Fig. 2, revealed an interesting pattern. While an increased number of time points improved model recovery when the within-cluster variance was low (especially in conditions with few persons, which needed a high number of time points to achieve satisfactory accuracy), this effect reversed when within-cluster variance was high $\textemdash$  leading to decreased model recovery in conditions with a high number of time points. To better understand this pattern, we investigated the over- and under-estimation of clusters by ICL for different conditions. When within-cluster variance was low (i.e., within-cluster standard deviations of 0.0018 or below), ICL never over-estimated the number of clusters (0% of times). However, once the within-cluster standard deviation increased to 0.0072, ICL frequently over-estimated the number of clusters (25.36% of times). This effect was further amplified by a higher number of time points and a higher number of persons. In contrast, under-estimation of the number of clusters remained relatively stable across different levels of within-cluster variance (see Table 3 in Appendix D). BIC and ICL performed virtually identical across all conditions. Thus, we report only the results of ICL and do not discuss BIC separately.

**Fig. 3:**
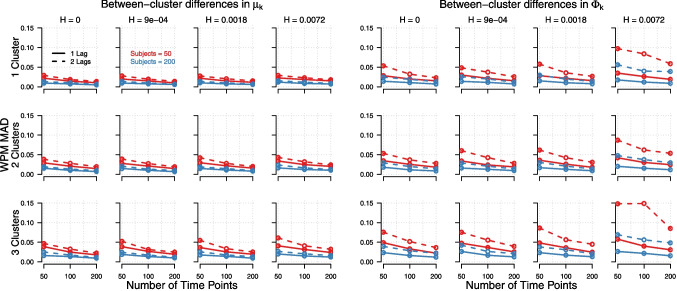
Mean absolute deviation between estimated and true parameters for the means, $\boldsymbol{\mu }_k$, as a function of the number of true clusters (*rows*), the within-cluster variance (*columns*), the number of time points (*x-axis*), the number of subjects (*red vs. blue*), and the number of lags (1 lag = *solid*, 2 lags = *dashed*). *Left panel*: When between-cluster differences were present in the means, $\boldsymbol{\mu }_k$. *Right panel*: The corresponding results when between-cluster differences were present in lagged effects matrix, $\boldsymbol{\Phi }_k$

**Fig. 4:**
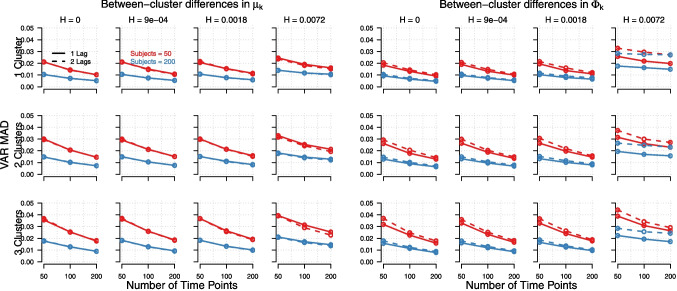
Mean absolute deviation between estimated and true parameters for lagged effects matrix, $\boldsymbol{\Phi }_k$, as a function of the number of true clusters (*rows*), the within-cluster variance (*columns*), the number of time points (*x-axis*), the number of subjects (*red vs. blue*), and the number of lags (1 lag = *solid*, 2 lags = *dashed*). *Left panel*: When between-cluster differences were present in the means, $\boldsymbol{\mu }_k$. *Right panel*: The corresponding results when between-cluster differences were present in lagged effects matrix, $\boldsymbol{\Phi }_k$

#### Estimation recovery

Regarding estimation recovery of the parameters, $\boldsymbol{\mu }_k$ was estimated more accurately in the following conditions: 1 lag models (compared to 2 lag models), fewer clusters, a higher number of persons, more time points, and lower within-cluster variance (see Fig. 3).

Estimation recovery was also higher when between-cluster differences were in $\boldsymbol{\mu }_k$ rather than $\boldsymbol{\Phi }_k$ (in contrast to model recovery, which was higher when differences were in $\boldsymbol{\Phi }_k$ rather than $\boldsymbol{\mu }_k$). The same main effects can be seen for the estimation recovery of $\boldsymbol{\Phi }_k$ (see Fig. 4). These main effects were consistent for both the autoregressive and cross-lagged parameters in $\boldsymbol{\Phi }_k$, although the recovery of autoregressive parameters was generally poorer than that of cross-lagged parameters (see Table 5 in Appendix D). This likely because the autoregressive parameters had higher true values in our simulation, meaning that although their absolute estimation errors were higher, their relative deviations (as a proportion of the true parameter value) are comparable.

Cluster membership recovery, measured by the ARI, was excellent across all conditions (according to guidelines by Steinley, 2004). In conditions with at least 100 time points, recovery was virtually perfect (see Fig. 12 in Appendix D). This indicates that the LCVAR model reliably classified individuals into the correct clusters across all simulated conditions. The main effects of the six simulation factors on estimation recovery are summarized in Table 4 in Appendix D.

### Discussion of simulation results

Regarding the recovery of the true underlying model by information criteria, we draw the following conclusions. Higher within-cluster variance especially decreased the recovery of the number of clusters with large within-cluster variance resulting in the number of clusters being over-estimated by ICL and BIC. This is an expected result because LCVAR does not account for within-cluster variation in a person’s individual model parameters; hence, any between-person differences can only be accounted for through additional clusters. This means that the ICL/BIC are in general inconsistent in the sense that in the presence of within-cluster variation, they will over-estimate the number of clusters, and estimate more clusters when there is more data. While we evaluate the behavior of ICL/BIC in the presence of this type of misspecification in situations resembling applied research, this in general limits the utility of these information criteria for model selection. Instead, in practice one uses a combination of many indicators to select a final model, as we will show in the tutorial section below.

Similarly, HQ’s accuracy in selecting the lag order combination decreased in conditions with higher within-cluster variance. For both, the recovery of the number of clusters and of the lag order combination, a higher number of time points and a higher number of persons amplified these adverse effects. Based on our simulation results, we recommend that empirical researchers use HQ rather than SC to select the lag order combination, unless the number of time points or persons is at least 200. In such cases, researchers should favor SC over HQ. Regarding the selection of the number of clusters, researchers can use either ICL or BIC; we found virtually no differences in their performance. They are likely to select the same number of clusters.

Regarding the accuracy of parameter estimation we can conclude the following. Estimation recovery improved considerably when the number of time points increased beyond 50. However, a similar improvement could alternatively be achieved by increasing the number of persons in the dataset. This highlights a compensatory relationship between the number of time points and the number of persons, as shown in Figs. 3 and 4. Therefore, we recommend that researchers use LCVAR with datasets where individuals are measured with approximately 50 effective time points, or ideally more. For high estimation recovery of parameters, studies with fewer effective time points should compensate by including a larger number of persons, and vice versa. The specific balance between the number of time points and persons required for accurate parameter estimation and cluster membership recovery ultimately depends on the within-cluster variance. While model recovery improved when between-cluster differences were in the lagged effects matrix ($\boldsymbol{\Phi }_k$) rather than in the means ($\boldsymbol{\mu }_k$), the opposite was found for estimation recovery (i.e., for parameters and cluster memberships). This effect is unexpected; we have no explanation for why differences in one type of parameter would lead to higher model recovery but not also to higher parameter recovery.

To correctly classify individuals into clusters, LCVAR requires a sufficient number of effective time points^9^ per person. In our simulation study, 50 time points were adequate across all conditions to achieve excellent cluster membership recovery. However, we observed that the number of effective time points needed for accurate cluster assignment depends on the level of within-cluster variance. Considering these findings, we suggest the following regarding the required number of time points. In empirical scenarios with very high within-cluster variance, more than 50 effective time points may be required for reliable cluster membership recovery.

## Tutorial: Analyzing emotion time-series with LCVAR model

Here, we show how to use the *ClusterVAR* R package (version 0.0.8) to estimate a series of LCVAR models to emotion time-series of multiple persons measured in an EMA study, perform model selection, and interpret the selected LCVAR model.

### The example dataset

We use the data of Grommisch et al. (2020), who investigated individual differences in the use of emotion regulation (ER) strategies in daily life using EMA to explore how the variety and combinations of ER strategies relate to well-being. Their sample included 179 adults aged 18 - 69 years. The EMA design consisted of measurements over a 21-day period during which the participants were prompted between 10 a.m. and 10 p.m. at intervals of $80 \pm 30$ minutes, resulting in approximately nine prompts per day.

During each EMA prompt, researchers assessed a number of momentary emotions and the use of emotion regulation strategies. In this tutorial, we focus on the measurements of the emotions Happy, Relaxed, Sad, and Angry. The emotion items asked how [emotion] participants felt at the moment at which the measurement was taken and the responses were scored on a Visual Analogue Scale ranging from 0 (not at all) to 100 (very much).

### Visualizing the time-series

**Fig. 5:**
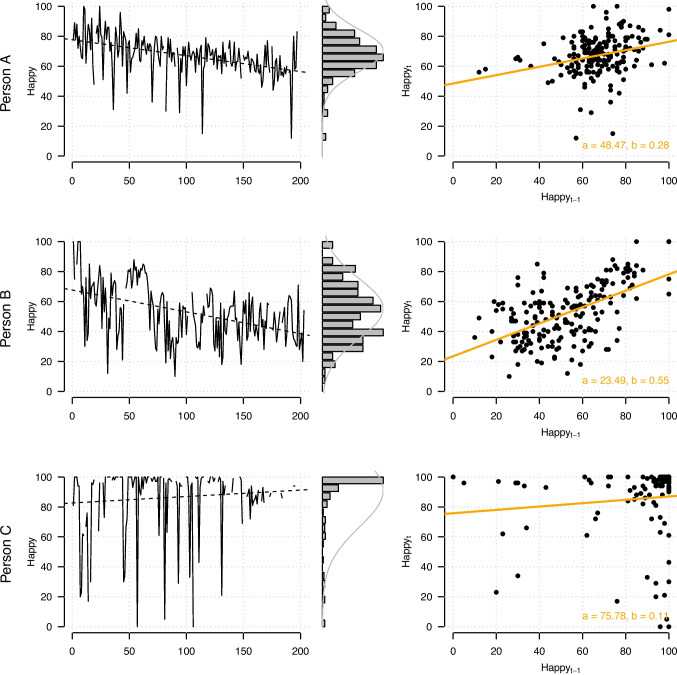
*Left*: Line plots of the variable Happy across all time points of the study including their marginal histograms for three randomly selected persons; the *dashed line* indicates the best-fitting linear time trend. *Right*: Scatter plot showing the relationship between $\text {Happy}_{t-1}$ and $\text {Happy}_{t}$ across all time points, for the same three persons. The *orange line* shows the best-fitting linear regression line to describe this relationship

Before fitting any model, we visually inspect the time-series data. Figure 5 shows line plots of the variable Happy over time (left panels) and the relationship between $\text {Happy}_{t-1}$ and $\text {Happy}_{t}$ (right panels) for three randomly selected persons A, B, and C (rows). Time-series plots for *all* variables and *all* persons can be found in our reproducibility archive, together with code to reproduce the entire tutorial: https://github.com/jmbh/LCVARTutorial. These data visualizations are insightful for two reasons.

The first reason is that inspecting the data allows us to judge whether a VAR model can in principle describe the time-series well. For persons A and B, we see marginal distributions that are still reasonably well approximated with Gaussian distributions (see histograms and best-fitting Gaussian density shown as gray line). However, for Person C, we see a high point-mass at the upper scale end (at 100), while the rest of the density is scattered across the rest of the scale. We already know that such time-series are modeled poorly by VAR models, since VAR models only generate unimodal Gaussian-shaped marginal distributions (e.g., Hamilton, 1994).

Looking at the relationship between $\text {Happy}_{t-1}$ and $\text {Happy}_{t}$, we see that the relationships can be reasonably well approximated with a linear function for persons A and B, as we do not see any obvious non-linearities. However, for person C, we see that the relationship is not well approximated by a linear function. This is because much of the measurements are at the scale end at 100 and the variation seems to follow a kind of switching behavior between 100 and other values across the entire scale. Overall, after inspecting the plots of all other variables across all persons, we conclude that there is substantial heterogeneity and potential misfit of the VAR model, but that the VAR model can still be a useful model to describe the dynamics in the multivariate time-series, especially when being able to assign persons to different VAR models as we do in the LCVAR model. We turn to this aspect next.

The second reason why inspecting the data is useful is to see whether a latent class model could in principle be a good model for the heterogeneity in VAR models between persons. Inspecting the three persons in Fig. 5 suggests that there is considerable heterogeneity both in the value of the mean and the autoregression of Happy. For example, we see that the strength of the linear relationship between $\text {Happy}_{t-1}$ and $\text {Happy}_{t}$ differs considerably between Person A and Person B. However, we also see that there does not seem to be a linear relation between these two variables at all for Person C. While in this case a LCVAR model cannot provide a good fit, it may still be a useful summary of the heterogeneity in the dynamics, because it may allocate all persons with a similar pattern as Person C (including high mean for Happy and close-to-zero autoregression $\text {Happy}_{t-1} \rightarrow \text {Happy}_{t}$) to the same cluster.

Of course, the extent to which heterogeneity can be assessed visually is limited. This is because we are interested in the heterogeneity with respect to all parameters of the VAR model, which we cannot visualize simultaneously. Thus, based on data visualization alone, it is difficult to determine the extent and structure of heterogeneity. For example, we cannot determine whether heterogeneity between persons is best modeled with a continuous distribution (i.e., a multilevel model with a latent Gaussian distribution) or discrete clusters (i.e., a LCVAR model).^10^ However, the data visualization in this dataset suggests that there is heterogeneity between persons that is unlikely to be due to sampling variation alone, which motivates using a model that captures heterogeneity between persons, such as the LCVAR model.

### Assessing non-stationarity

Detecting systematic changes in time-series, such as trends (e.g., systematic increases in the mean of a variable over time), is important for two reasons. First, such changes are almost always of substantive interest. This is especially the case when trends can be linked to changes in the environment of the person. These changes could be contextual (e.g., working vs. holiday), natural experiments (e.g., COVID-19 lockdown), or planned interventions (e.g., treatments of disorders). However, even when changes cannot be linked to environmental factors, it is often of substantive interest to know how a variable of interest systematically changes across time.

The second reason why modeling trends is important is that if trends are present in the data but are not modeled, they bias the remaining parameters in the model. For example, if we generate data from a model with a linear trend and no dependency between measurements beyond the trend, and then fit an autoregressive (AR) model without a trend to these data, we obtain large positive estimates for the autoregression, even though this parameter is zero in the data-generating process. Consequently, interpreting the resulting high autoregression as a strong causal effect or as high stability of the variable would clearly be incorrect.

What can we conclude about mean trends based on visually inspecting the time-series of the three persons in Fig. 5? In the time-series of Person A, the mean seems to decrease relatively linearly over the period of the 21 days. This is confirmed by fitting a linear trend to the data, which is displayed as the dashed black line. For Person B, we also see a negative trend; however, here we see that the linear trend is not capturing the trend as well as for Person A, since we see considerable fluctuations around the dashed linear trend. For Person C, Happiness seems to improve over time. However, we see that while a linear time trend picks up this tendency, it does not capture the underlying pattern well: instead of a slow movement of the mean, we see that the higher values towards the end of the time-series is because the person does not “switch” to very negative states any more.

One approach to model trends in the mean is to estimate them separately for each univariate time-series, subtract them from the data (i.e., “detrend” the data), and then model the dynamics in the detrended data. However, this approach has several downsides. First, the uncertainty in the estimation of the trend is not propagated into the model for the dynamics (e.g., McElreath, 2018, Chapter 5). Second, we cannot investigate any heterogeneity in trends across persons because we already removed them from the data before fitting the LCVAR model. To avoid these issues we include a linear time trend in our LCVAR model. These time trends are allowed to vary across clusters and therefore estimated clusters can be different in terms of their time trend. We have seen in Fig. 5 that the *linear* trend does not capture all trends well. This suggests that some remaining non-linear trends will still affect our estimates. However, distinguishing non-linear deterministic trends from noise is an inherently different task (e.g., Haslbeck et al., 2021) we will not focus on in this tutorial.

In the present dataset, the time-series of persons are not aligned on a common time-axis and therefore do not share a common environment whose changes could systematically be linked to changes in persons. This would be different if the environment of the modeled persons was aligned and we had information about what happened in that environment. For example, we might have measured people in the Netherlands starting on May 1st 2020; on May 15th the government issued a nation-wide lockdown due to COVID-19, which likely substantially affected all persons in the study. We can then see whether there are clusters that differ in how their measured variable’s means are affected by the COVID-19 lockdown. Another example could be a planned treatment for a mental disorder and different responses to that treatment. The fact that group-level trends are only meaningful if environments are aligned is true for all hierarchical models including the multilevel VAR model. The motivation for including a linear time trend for the mean in our LCVAR example is therefore primarily to avoid bias in the lagged effects.

So far, we have discussed only linear trends of the mean, but trends can also be non-linear and cyclic (Haqiqatkhah & Hamaker, 2024). For example, the happiness of a person might be higher during the weekend compared to week days, or during the morning compared to the evening. One way of modeling such trends on the mean would be to use dummy-coded covariates, which is also possible in the *ClusterVAR* package. For an example of an LCVAR model that includes such cyclic trends see Ernst et al. (2020). However, not only the mean can change over time, but also the variance, lagged effects, or higher-order moments of the distribution such as skewness. These types of non-stationarity have so far been barely explored in EMA time-series and represent an interesting avenue for future research. The current version of *ClusterVAR* does not accommodate changes across time in these parameters, only changes in the mean. For a gentle introduction to non-stationary time-series, different forms of non-stationarity, and a discussion of how to detect it in empirical data, see Haslbeck et al. (2021); Ryan et al. (2024); Zhang et al. (2024).

### Missing data handling

The implementation in LCVAR() $\textemdash$  the function from the *ClusterVAR* package that estimates LCVAR models $\textemdash$  will exclude all data from a time point if any data at that time point is missing. Additionally, LCVAR() only uses a time point for estimation when the required earlier time points are observed. For example, if we fit lag 1 models, we only use those time points for which the previous time point is also available. If we fit a lag 2 model, we only use those time points for which the previous two time points are available (see also Section “Lag orders and the effective number of time points” on effective number of time points).

To assess which time points meet these requirements, the user provides indicators for the day and the notification during each day (see below). Hence, because LCVAR() does not predict time points that cannot be predicted, it is no longer necessary to impute missing data before using the LCVAR() function, in contrast to previous implementations of the function (Ernst et al., 2020). Separate from the estimation function, the function |numberPredictableObservations()| allows users to compute the number of effective time points for a dataset given a specified lag. For our example dataset, this shows that for a lag 1 model, we can use 22,931 (77.9%) of the total 29,431 time points (rows in the dataset). For a lag 2 model, we could only use 60.8% of the time points for estimation.

The approach of including only predictable time points in the estimation effectively treats certain observations as missing. While this results in the exclusion of some time points, it has the advantage of ensuring that the remaining data are equally spaced. This is important because applying a VAR model to unequally spaced time-series data can introduce bias into the parameter estimates (de Haan-Rietdijk et al., 2017).

### Fitting sequence of LCVAR models

We now explain how to specify the |LCVAR()| function from the *ClusterVAR* package. We provide the data matrix via the argument |Data| and specify the column numbers of the variables that should be modeled (|yVars|), the unique identifier for each person (|ID|), and the variables indicating on which day (|Day|) and on which measurement occasion (|Beep|) a given measurement was taken. This latter information is used to ensure that only time points with sufficient previous time points are used for estimation (see Section “Missing data handling”). When an argument is supplied for |Day|, the first measurement of a day is not predicted based on measurements on the previous day. Thus, in datasets where there is only one measurement per day, no argument should be supplied for |Day|.
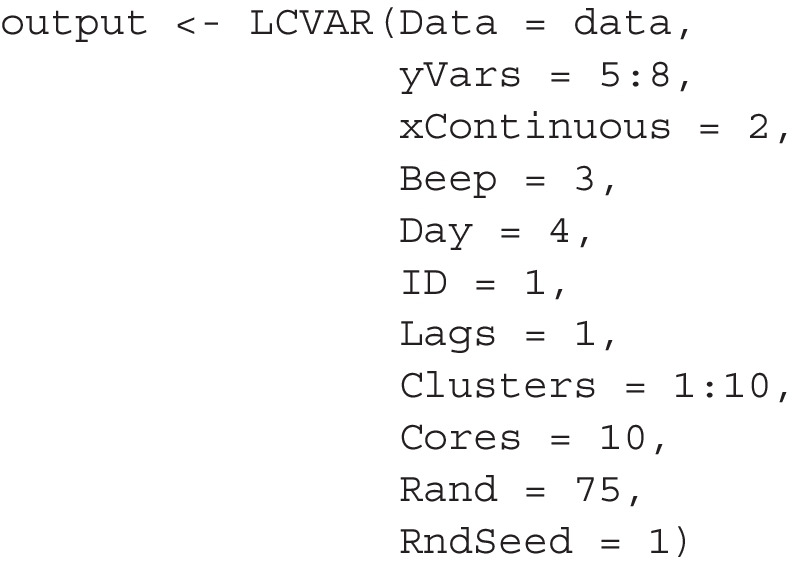


The |Lags| argument specifies the number of lags that are considered for each cluster. Here, we set |Lags = 1| so that the VAR models in each cluster have only a lag of 1. We make this choice to avoid substantial loss of data when including a lag of 2 (see Section “Lag orders and the effective number of time points”).

The |Clusters| argument specifies the sequence of models with different numbers of clusters *K* fitted to the data. How should one select this sequence in practice? Sometimes we have theoretical expectations about how many clusters might be a good description of the data. If we expect $K=2$ clusters, a natural sequence would start at $K=1$, increase to the expected number of clusters, and include one additional cluster, resulting in $K\in \{1,2,3\}$ in this case. If no prior expectations exist, a purely data-driven approach can be used, starting with a sequence from $K=1$ up to an arbitrarily chosen *K* (e.g., $K=10$) and assessing the fitted models. If the highest included *K* turns out to still be an interesting model, one may choose to extend the sequence by estimating additional models. Since we have no strong theoretical expectations, we specify a sequence of $K \in \{1, 2, \dots , 10 \}$. We chose a maximum $K=10$ because, in the best-case scenario for estimation with an equal distribution across clusters, this results in $\frac{179}{10} \approx 17.9$ persons per cluster with $K=10$, which we consider relatively small for obtaining precise parameter estimates.

The argument |xContinuous| allows one to specify continuous covariates that explain trends in the mean. As discussed above, we include a linear trend to the model, which we do by specifying |xContinuous = 2|, because the second column in the dataset is a vector running from 0 to the total number of rows (including missing time points) for each person. Defining a time variable that begins with 0 as covariate allows us to interpret the estimated means as the mean at the beginning of the time-series (for an interpretation of the mean, see Section “LCVAR model estimation”). We can also add multiple continuous covariates at |xContinuous| or add (nominal) categorical covariates, the latter must be specified with the argument |xFactor|. For instance, by including a categorical variable indicating the time of day, this model can estimate a different mean for the different times of day (Bringmann et al., 2024; Ernst et al., 2025). See Ernst et al. (2020) for an example of such a model.

The argument |Cores = 10| specifies that we run the estimation function in parallel on 10 cores. |Rand = 75| specifies that we use 75 random restarts of the EM algorithm to avoid local maxima. Finally, |RndSeed = 1| sets a random seed to ensure that the analysis is reproducible despite parallel computing.

Users can supply additional arguments for the initialization, the number of iterations for the EM algorithm, the convergence criteria, and standard arguments allowing one to switch into verbose mode or switching off the shown progress bar. If the argument |Center| is set to |TRUE|, all variables are within-person centered. This removes all between-person differences in means and therefore the clustering will only be based on differences in the lagged regression parameters of the VAR model. Above, we chose the default option of not centering the data, which means between-person differences in the means will influence the assignment of persons into clusters.

Just like for a regular VAR model, estimating the LCVAR model requires some variation in each variable of each person. In empirical data, this is sometimes not the case, especially in short time-series, for example, for strongly worded negative affect items, which are then only responded to with the same category at all time points. Because LCVAR cannot be estimated reliably when these cases occur frequently, it is important to be aware of these zero-variance time-series (for a technical explanation, see Bishop, 2006, pp. 430–349). Accordingly, the |LCVAR()| function displays warning messages indicating when this occurs, specifying the affected variables and persons. Generally, we advise users to specify |options(warn = 1)| before running the |LCVAR()| function, to ensure that warning messages are shown as they occur and are not suppressed.

### Evaluating sequence of fitted LCVAR models

We now inspect and compare the sequence of fitted LCVAR models with different numbers of clusters *K*. Our goal is to determine which of the fitted models provide a good description or representation of our data. To select a final model with a given number of clusters, we caution researchers against relying solely on information criteria. Our simulations showed that criteria such as the BIC and ICL tend to overestimate the number of clusters in LCVAR models when the data exhibit within-cluster heterogeneity, a situation that is likely in many empirical applications. For this reason, we recommend using information criteria only in combination with other strategies. In the following, we discuss several complementary strategies that can be employed alongside information criteria to select the number of clusters.

#### Convergence checks

Before inspecting any results, we assess for each of the 10 LCVAR models whether the EM algorithm converged to a solution as defined by the specified convergence criterion. Within the algorithm, multiple sets of starting values are generated to avoid local maxima, meaning, for each number of clusters *K*, we fit many LCVAR models and only consider the model with the lowest negative log likelihood. That is, for each number of clusters, we fit a number of models (this number is specified with the |Rand| argument) with different starting values and each start terminates either because it reached the maximum number of allowed EM-iterations (this number is specified with the |it| argument), or because the start reached the convergence threshold (this threshold is specified with the |Conv| argument). In the former case we consider the start as non-converged, in the latter case we consider the start as converged. Of all starts for a given *K*, we consider that start with the lowest negative log likelihood (i.e., best model fit) as the final solution for the given *K*. Here we only check the convergence of this winning model for each number of clusters. The convergence of this model is indicated in the summary of the fit object |summary(ouput)|. The summary shows that all 10 models converged, which means that we can consider all of them moving forward.

In practice, if many of the winning models for the different number of clusters have not converged, this might be a sign that an empirical researcher should increase the number of starts (specified with the |Rand| argument) and should particularly increase the maximum number of allowed EM-iterations (specified with the |it| argument). However, this will increase the necessary computation time of the |LCVAR()| function considerably.

#### Overall model fit and fit-indices

**Fig. 6:**
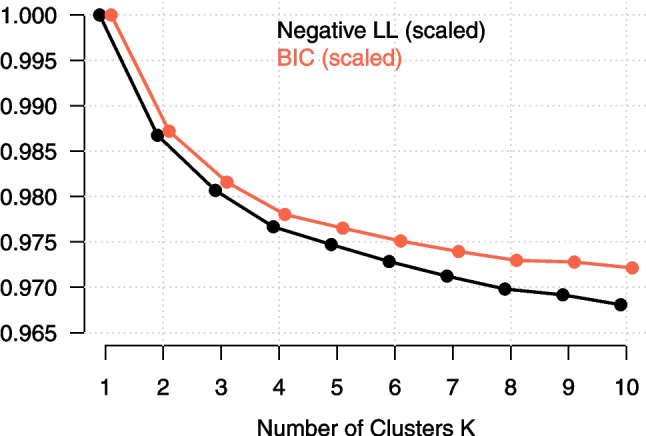
The negative log likelihood (*black*), the BIC (*orange*) for LCVAR models with $K \in \{1, 2, \dots , 10 \}$ classes (*x-axis*). Since the scale of the negative log-likelihood and the BIC are not meaningful and to be able to display all results in one graph, we scaled the negative log likelihood and the BIC by dividing each value by their respective maximum value

To get a first overview of how well the different models fit and how this fit trades-off with model complexity, we inspect the negative log likelihood and the BIC for each *K* in Fig. 6. We are looking at the *negative* log likelihood and therefore lower values indicate better model fit. Since we evaluate the within-sample model fit of increasingly complex models, we know that this line must decrease monotonously with increasing *K*. However, it is informative how exactly the line decreases: We see that there is a steep increase in model fit (i.e., decrease in negative log likelihood) when going from $K=1$ to $K=2$. When increasing *K* further, we see that the model fit increases further, but the increases become smaller and smaller as *K* increases. Yet, the increases in model fit decrease quite slowly and do not seem to have “flattened out” even at $K=10$.

**Table 1:** Proportion of persons in each of the clusters (columns) for models with different numbers of Clusters *K* (rows). The 1 cluster solution is not shown since every person is in a single cluster

	C 1	C 2	C 3	C 4	C 5	C 6	C 7	C 8	C 9	C 10
2 Clusters	0.45	0.55								
3 Clusters	0.42	0.48	0.09							
4 Clusters	0.35	0.28	0.09	0.28						
5 Clusters	0.08	0.16	0.34	0.15	0.28					
6 Clusters	0.13	0.08	0.20	0.15	0.18	0.26				
7 Clusters	0.17	0.15	0.17	0.17	0.13	0.08	0.13			
8 Clusters	0.13	0.12	0.06	0.10	0.20	0.15	0.11	0.13		
9 Clusters	0.15	0.13	0.05	0.12	0.15	0.08	0.11	0.14	0.07	
10 Clusters	0.17	0.12	0.11	0.20	0.13	0.04	0.06	0.11	0.03	0.04

In order to select a model that generalizes well to new samples from the same population, we need to trade off model fit with model complexity. For this purpose, the latent class and mixture modeling literature typically uses information criteria such as the BIC or the ICL (Biernacki et al., 2000; Nylund et al., 2007), which we evaluated in our simulation study. In Fig. 6, we only show the BIC, since it is virtually the same as the ICL, similar to our simulation results. We see that the BIC also decreases monotonously and is also minimized by $K=10$, although it flattens out earlier, from $K=4$ or $K=5$.

What can we take away from these results? It is clear that the heterogeneity is not well described by a small number of highly homogeneous clusters, because our simulation study showed that in that case, we would see a U-shaped path in Fig. 6, where the BIC decreases until a certain number of clusters, and then increases again. However, our simulations have also shown that if there is considerable heterogeneity within clusters, and the sample size is large, like in the present dataset, the BIC overestimates the number of classes.

In summary, the convergence checks did not rule out any of the models and the model fit and BIC suggest that model fit improves considerably from $K=1$ to $K=2$ and until around $K=4$, but when including additional clusters after that, there is no clear point at which the incremental improvement in model fit flattens out.

Commonly, in the clustering literature, model fit is not the only consideration when selecting the number of clusters *K* (Ram & Grimm, 2009). Generally, researchers want to select the best or most reasonable representation of the data. In what follows we discuss a number of additional considerations for selecting the number of clusters, based on the estimated parameters of the model and how they are aligned with the research goals at hand.

#### Distribution of persons across clusters

One additional way to select between different number of clusters *K* is to consider the proportion of persons assigned to each cluster across the various models. If in a given model, one cluster includes only a very small proportion, one may choose to exclude that model because its interpretation is less meaningful. For example, if we have a model with three clusters, which have proportions 0.45, 0.43, and 0.02, we might find the third cluster not large enough to have a meaningful interpretation and hence exclude the model with $K=3$ from our candidate models. We can inspect the distribution of persons across clusters in the output of the function |summary(output)| and summarize them in Table 1.

Considering that an even distribution of clusters would lead to a proportion of $\frac{1}{K}$ per cluster, we see that the persons are distributed relatively equally across the clusters for any *K*. Hence, we do not exclude any of the models for a certain number of clusters from our list of candidate models.

**Fig. 7:**
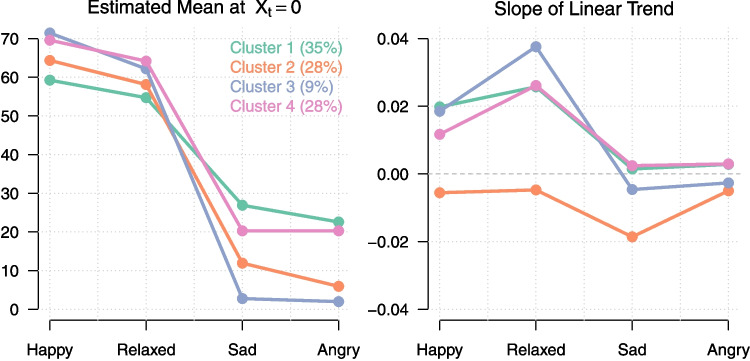
*Left*: The estimated cluster-specific mean at the beginning of the time-series for each of the four modeled emotion variables in each of the four clusters. The percentages indicate the prior probabilities for each cluster, which are also shown in Table 1; *Right*: The cluster-specific slope of the linear trend for each variable in each of the four clusters

#### Inspect cluster-specific parameter estimates

When selecting the number of clusters, another central consideration is the cluster-specific parameter estimates associated with a given number of clusters *K*. If parameter estimates for a given *K* were impossible or implausible, we could reject a model for that reason. For example, we might observe that estimated means are outside the empirical measurement scale or that the lagged effects matrices are not stable (i.e., eigenvalues are larger than 1 in absolute value). However, in most empirical cases the parameter estimates will be plausible enough and we instead have to make more nuanced and substantive judgments. This can be based on looking at the cluster-specific estimates for different *K*s and judging up to which point adding more clusters creates additional meaningful insight. For example, we might have a model with $K=4$ clusters and are considering adding one more cluster. If in the model with $K=5$ clusters the parameter estimates of some clusters are similar to one another, or if a new cluster dimensionally falls between two existing clusters from the $K=4$ model, we may decide to choose the simpler $K=4$ model. In practice, researchers can also use empirical justifications based on existing theories and literature to judge which model provides the most reasonable/expected representation of the data.

In practice, one would inspect the cluster solutions for all considered *K* to see whether parameter estimates are sensible and whether they provide a useful description of the data. In the interest of brevity, we here only discuss the model with $K=4$ clusters as an example for how cluster-specific model parameters can be displayed and interpreted, both for selecting *K* and for presenting a final model. For a discussion of how the interpretation of LCVAR model parameters differs from that of other popular VAR-based models, see Appendix A.

We first consider cluster-specific means and linear trends. Because we included a linear trend, the mean estimate can be interpreted as the mean at the first observation of the time-series (i.e., at $x_t = 0$). The means at the beginning of the time-series are shown in the left panel of Fig. 7. For Clusters 1, 2, and 3 we see that means of positive and negative emotions are negatively correlated: the higher the means of two positive emotions, the lower the means of the two negative emotions. However, this is not the case for Cluster 4: here, we see that the means of both positive and negative emotions are among the highest compared to the other clusters. Importantly, such patterns are difficult to capture with a dimensional model for heterogeneity (i.e., the multilevel VAR), both because the different correlations between positive and negative affect means are shrunk towards an overall correlation, and because that model does not explicitly identify persons that fall into this cluster. The results could either indicate that Cluster 4 contains persons who feel their emotions very strongly regardless of the valence (i.e., persons in Cluster 4 exhibit distinct emotion dynamics), or persons who are prone to embrace the extreme ends of a scale when filling in a questionnaire (i.e., persons in Cluster 4 exhibit a distinct response style (Henninger & Meiser, 2020)).

We next consider the cluster-specific time trends. We see that the slopes vary between around $-0.02$ for Sad in Cluster 2 and 0.04 for Relaxed in Cluster 3. Since the time-series are about 200 time points long (see Fig. 5), this corresponds to a maximum increase in the mean of $0.04 \times 200 = 8$ points on the [0, 100] scale. As discussed in Section “Assessing non-stationarity”, in the present dataset the environments of the persons were not time-aligned and we do not know of any events that could explain trends in any of the variables. The estimated slopes are therefore considered nuisance parameters in this model, which are only estimated to avoid bias in the estimates of the lagged effect parameters. In a dataset in which all persons are starting at the same time and share an environment in which, for example, a heatwave occurs, or a treatment is being administered, the slopes would be much more meaningful and could be interpreted as responses to these changes in the environment. The only way in which the persons are aligned in our study is that they all start an EMA survey on their first day. The fact that the slopes are generally small indicates that there is no consistent increase or decrease of emotions across people within a cluster. Hence the mean values at the first observation, which are shown in Fig. 7 more or less describe the means of the emotions across the whole time-series.

**Fig. 8:**
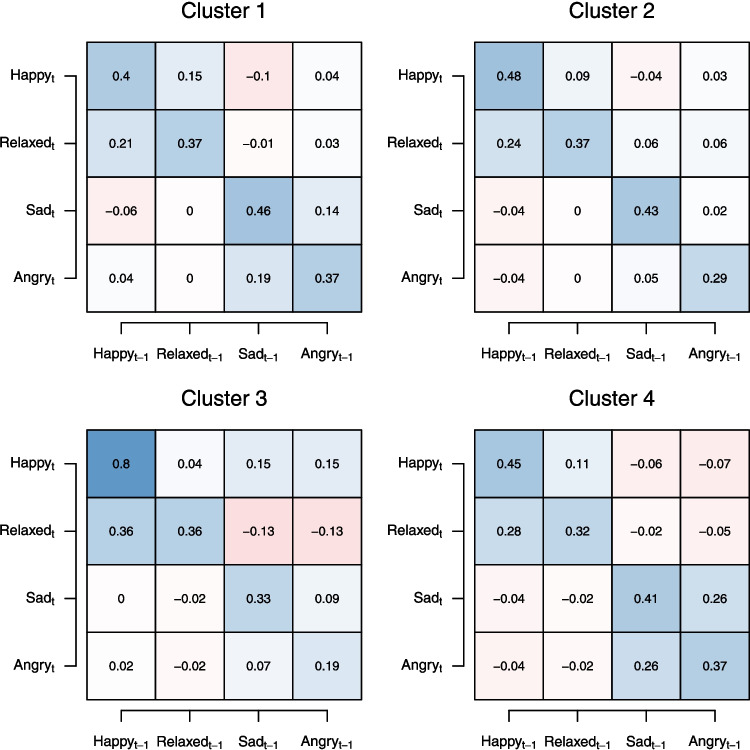
The cluster-specific autoregression and cross-lagged regression estimates in each of the four clusters of the lag 1 model with K = 4

We next consider the cluster-specific lagged effect parameters for each cluster, which are shown in the four panels of Fig. 8. We see that the autoregressions in the diagonal are all positive and larger than the cross-lagged regressions in all clusters. This makes sense and is in line with common results of VAR models estimated on emotion time-series (Ryan et al., 2024). We also see that cross-lagged regressions within valence tend to be positive and relatively large compared to the cross-lagged regressions between valences, which tend to be negative and relatively small. Inspecting differences between clusters, we find clearly distinct patterns: Cluster 3, which has the highest mean of Happy and the lowest means for Sad and Angry of all clusters, is characterized by the highest autoregression (i.e., stability) for Happy, showing that the persons whose mean Happiness scores are highest also have the most stable Happiness scores. We also see that $\text {Happy}_{t-1}$ has a big effect on $\text {Relaxed}_{t}$ but not the other way around, and there is a positive effect of both $\text {Sad}_{t-1}$ and $\text {Angry}_{t-1}$ on $\text {Happy}_{t}$. In particular, the positive effect of both $\text {Sad}_{t-1}$ and $\text {Angry}_{t-1}$ on $\text {Happy}_{t}$ seems counter-intuitive and thus requires post hoc inspections and analyses to determine the time-series patterns underlying these results. Cluster 1, which has the lowest means on the positive emotions and highest means on negative emotions, is characterized by high autoregressions for Sad and Angry and the lowest autoregression for Happy. Also, for all emotions, autoregressions are roughly equal to around 0.40, cross-lagged regressions of emotions with the same valence are moderately high, but cross-lagged regressions across valence are close to zero.

Cluster 2, which is average in the means of all variables, is characterized by autoregressions similar to Cluster 1 and 4, but with cross-lagged regressions that are close to zero, with the cross-lagged regressions between Happy and Relaxed being the only exception. Finally, in Cluster 4, which stood out for exhibiting high means on positive as well as negative emotions, we see again autoregressions around 0.40, stronger positive cross-lagged regressions within valences, and smaller, but consistently negative cross-lagged regressions between valences. In this cluster, it is especially notable how strong the cross-lagged regressions of Sad and Angry are.

An inspection of the cluster-specific mean and lagged effect estimates across clusters in the model with $K=4$ suggests meaningful differences between clusters. However, since data visualization has already shown that the VAR model is severely misspecified for some individuals, we know that the parameters do not tell us everything about the persons in the four clusters. To ensure that we interpret the clusters and their parameters adequately, we recommend performing a residual analysis in which we compare observed values to their predictions based on the cluster-specific parameter estimates, to see whether certain patterns (of misspecification) are occurring in certain clusters and if model fit systematically differs across clusters.

#### Residual analysis

Like for any statistical model, we can assess the fit of the model to the observed data by comparing the predicted values with the associated observed data. This allows us to compute how well different variables can be predicted over the specified time lag and how this differs between clusters and persons. Residual analysis (i.e., of innovations $\boldsymbol{u}_{i\,t}$) can also help identify systematic misfit, such as trends or a clear switching behavior, which are left unexplained by the fitted model.

In Appendix F, we compare predicted values with observed values for the time-series of Happy for the same three persons as in Fig. 5, using the model with $K=4$. We also compute the proportion of explained variance ($R^2$) for every variable and person and summarize it across variables and clusters. The latter analysis shows that, for example, the average $R^2$ (across variables and persons) over the approximately 80 minutes between measurements in the present study is about 12.60%. Such information should be taken into consideration when drawing theoretical conclusions from the fitted model.

#### External validation

Yet another way to select between models with different *K*, or to externally validate a given selected model, is to relate the estimated cluster membership of persons with between-person variables of interest. If we study individual dynamics, such as emotion regulation in daily life as in the study of Grommisch et al. (2020), we may be interested in between-person measures of outcomes of the studied dynamics. One type of measure that is often of interest is capturing experienced problems or symptoms of mental disorders. In their study, Grommisch et al. (2020) assessed the DASS-21 at baseline. The DASS-21 consists of three 7-item subscales measuring depression, anxiety, and stress (Henry & Crawford, 2005).

Here, we will relate the cluster membership of the $K=4$ cluster solution to these three subscales. Specifically, we classify each person into the cluster for which they have the highest membership probability. We use the R package *brms* (version 2.22.0; Bürkner, 2017) to compute a Bayes Factor $\text {BF}_{10}$. This compares $\text {H}_0$, which states that the means of a given DASS-21 subscale are the same across the four clusters, and the alternative hypothesis $\text {H}_1$, which states that the means are not the same. Therefore, $\text {BF}_{10} > 1$ indicates evidence for $\text {H}_1$. For the depression subscale we find $\text {BF}_{10} = 1496$, for the anxiety subscale $\text {BF}_{10} = 3909$, and for the stress subscale $\text {BF}_{10} = 56168$. Using Jeffreys’ rules of thumb Jeffreys (1961) for interpreting Bayes factors, we interpret $\text {BF}_{10} = 1-3$ as weak evidence, $\text {BF}_{10} = 3-10$ as moderate evidence, $\text {BF}_{10} = 10-30$ as strong evidence, $\text {BF}_{10} = 30-100$ as very strong evidence, and $\text {BF}_{10} > 100$ as extreme evidence for $\text {H}_1$. Consequently, we can very confidently conclude that the means of all three subscales differ across the four clusters. These results suggest that cluster membership is meaningfully associated with depression, anxiety, and stress.

It is perhaps not too surprising, that the depression, anxiety, and stress scores measured by the DASS-21 differ across cluster memberships, since the LCVAR models contain a cluster-specific mean parameter and the modeled variables are Happy, Relaxed, Sad, and Angry, which we intuitively would think are related to the baseline DASS-21 measures. It is therefore useful to examine how much the DASS-21 subscales still differ across clusters after conditioning on $\boldsymbol{\mu }_k$, the cluster-specific means of the four time-series variables. The resulting Bayes factors are $\text {BF}_{10} = 66$, $\text {BF}_{10} = 4$, and $\text {BF}_{10} = 42$ for depression, anxiety, and stress respectively. This shows that there is still moderate to very strong evidence for group differences across the four clusters in each of the scales above and beyond what could be explained by the means of the modeled variables.

**Fig. 9:**
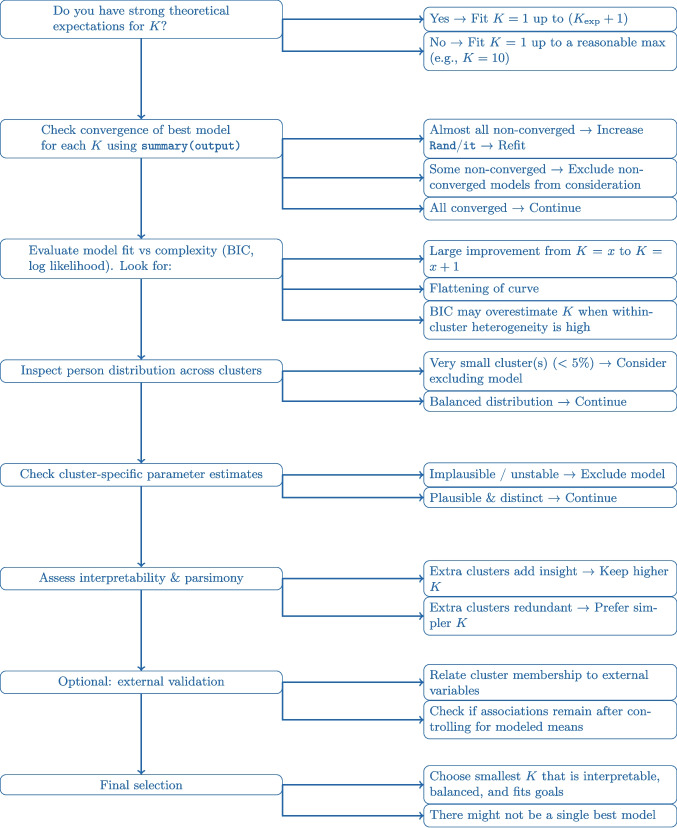
Decision tree for selecting a final number of clusters *K*. A researcher starts at the top of the diagram with a number of candidate models all with different number of clusters and excludes/selects candidate model(s) at each subsequent step

#### Conclusions: Selecting the number of clusters

Here we summarize the above results and discuss what we can conclude from them for selecting a model with a number of clusters that provides a good description or representation of the data. We summarize our advice on selecting the number of clusters in a decision tree in Fig. 9.

All models converged and were therefore considered for further analysis. The model fit and BIC as a function of *K* showed large improvements going from $K=1$ to $K=2$, and generally increased up to at least $K=4$. After that, improvements became smaller with increasing *K*, but they did not level off even at the maximum considered $K=10$. The results are consistent with a situation in which there is large heterogeneity between persons, so that additional clusters keep on improving the model fit. This is also consistent with inspecting the data visually, which revealed high heterogeneity between people. However, we do not select $K=10$ as the final model, because we are not only interested in model fit, but also in obtaining a model that is parsimonious enough to be understandable and to be communicated to other researchers.

What does this tell us about the structure of heterogeneity between individuals? From our simulation results we know that if the population consists of a small number of highly homogeneous $K^*$ subgroups, then the BIC correctly selects $K^*$ clusters. However, we also saw in our simulation that in the presence of considerable within-cluster heterogeneity, the BIC over-estimated the number of clusters. The analysis of model fit and BIC, together with the visualizations and the simulation results, suggests that more than $K=2$ clusters are needed. However, BIC does not indicate a clearly superior model between the remaining models $K \in \{3, 4, \dots , 10\}$.

In practice, empirical researchers likely have theoretical expectations regarding group structures, which can aid their selection of a cluster solution which is substantively interesting and likely provides a reasonable representation of the underlying data. In the tutorial above, we inspected only the parameters of the model with $K=4$ clusters and found differences between clusters that a dimensional heterogeneity model could not capture. This suggests that the clustering model may provide additional insights compared to a dimensional multilevel VAR model. We did not compare the parameters of all models for $K-$selection as we aimed to keep the tutorial concise.

We then related cluster membership with external between-person measurements for the model with $K=4$. We saw that cluster membership was related to all three subscales of the DASS-21, and this relationship persisted even when controlling for the means of all modeled variables, though we were less confident in its strength. Finally, we examined how persons were distributed across clusters for all models $K \in \{1, 2, \dots , 10\}$. Since the proportions per cluster were relatively balanced in all models, this did not provide strong evidence to exclude any specific model.

In summary, the results are consistent with considerable heterogeneity across persons and suggest that at least $K=3$ or $K=4$ are needed to provide good model fit, when estimating an LCVAR model on these data. We focused on the model with $K=4$ clusters to show how parameter estimates can be interpreted and that, with more background knowledge, these interpretations could be used to select an adequate number of clusters *K*. Also, for the model with $K=4$ we showed how models can be validated by relating estimated cluster membership to external criteria of interest. Combining the inspection of parameter estimates and external validation for several *K* together with background knowledge specific to the research at hand, may allow one to narrow the models down to only a few or even one model that provides a good description of the data and addresses the research goals at hand. However, in some cases $\textemdash$  perhaps such as in our tutorial $\textemdash$ it may not be possible to definitively identify a single best model. This, of course, is also an empirical result that can be used to inform theorizing about the topic of interest.

While the estimated LCVAR models suggest that there is considerable heterogeneity between persons and that at least some of that heterogeneity is well captured by the categorical latent variable in the LCVAR model, we did not perform model selection between the LCVAR model and other models for heterogeneity between people such as a dimensional multilevel VAR model. We revisit this consideration in the Discussion section.

## Discussion

The aim of this paper was to provide psychological researchers with everything they need to use LCVAR models to analyze their time-series data. We have provided an accessible yet comprehensive introduction into LCVAR models, their estimation, and information criteria which can be consulted for the selection of an LCVAR model. To help researchers determine when LCVAR models can be reliably estimated from empirical data, we conducted a simulation study that investigated the performance of the LCVAR and its information criteria in scenarios resembling empirical research designs. Finally, we provided a comprehensive, fully reproducible tutorial on analyzing empirical data using the LCVAR model with the new *ClusterVAR* R package (Ernst & Haslbeck, 2024), covering data preprocessing, model fitting, model selection, and parameter interpretation.

### Implications for applied researchers

We evaluated the LCVAR model in a simulation study in which parameters and individuals’ cluster memberships were accurately estimated across a number of empirically realistic settings. The information criteria we evaluated in our simulation for selecting the lag order combination or the number of clusters, recovered the underlying LCVAR models well, as long as the within-cluster variance was small. When within-cluster variance was high, information criteria tended to over-estimate the number of clusters. In this section we first discuss how researchers can select among a number of candidate models, then we summarize requirements for the sample based on our simulation study.

#### Model selection with heterogeneity within-clusters

In any clustering application the selection of the final model among a set of candidate models is an essential and challenging task. Here, we summarize first the selection of the number of clusters when the lag order is fixed, then the simultaneous selection of the number of clusters and the lag order.

In our simulation we showed that because LCVAR does not account for within-cluster variance, information criteria, like the ICL and the BIC, consistently over-estimate the number of clusters in situations where the within-cluster variance is large. This is what we would expect, because the only way for the LCVAR model to account for additional population heterogeneity is to model it with additional clusters. This is also in line with the finding that large overlap between clusters can lead to an overestimation of the number of clusters by information criteria (Ding et al., 2021). Since most empirical EMA data likely exhibit within-cluster variance to some extent, one should not select the number of clusters on information criteria alone. Instead, it is crucial to outline strategies for how to select a final model that describes the underlying data well despite (large) within-cluster variance.

In our tutorial we therefore discussed a number of strategies that can be combined to select a final model with a specific number of clusters, even if information criteria continue to decrease because of high within-cluster variance or because the heterogeneity is generally structured in a more dimensional way. For instance, we encourage researchers to use empirical justifications for which model provides the most useful representation. We also compare models by illustrating each model’s practical significance and clarifying the value of the clusters identified by discussing covariates of the classification. In addition, we take into account differences in average parameters between clusters for each of the candidate models. These strategies are commonly employed in the clustering literature (Nylund et al., 2007; Ram & Grimm, 2009) to select the model which provides the best or most reasonable representation of the observed data.^11^ We also consider how persons are distributed across clusters and recommend excluding models with clusters that contain only a very small proportion of people. In practice, researchers can also use split-half internal cross-validation to assess model stability and aid in selecting the number of clusters (Heylen et al., 2016; Krieger & Green, 1999; McIntyre & Blashfield, 1980), though we have not considered this option in our tutorial section.

In our simulation and tutorial sections we showed that despite unaccounted variation within-clusters, LCVAR can classify persons in a meaningful way and estimate useful cluster averages. Thus, even if clusters are not entirely homogeneous, LCVAR can still produce meaningful clusters that provide a useful summary of the observed data.

#### Simultaneous selection of the number of clusters and lag order combination

We discussed that information criteria cannot select the lag order combination and the number of clusters simultaneously, thus, one of these two always has to remain fixed while selecting the other. In our tutorial, we fixed the lag order for all clusters to 1 while selecting the number of clusters, as this lag order is the most feasible for estimation on most EMA data due to missing data and the day-night break. In Section “Lag orders and the effective number of time points”, we elaborate that a higher lag order usually leads to a high loss of data during estimation in empirical datasets that contain missing data and night gaps. In case researchers want to nevertheless select the lag order in addition to the number of clusters, we recommend that they first select the number of clusters while fixing the lag order to 1 across all clusters. Then, in a subsequent step they can fix this resulting number of clusters while selecting the lag order combination across clusters through one of the time-series information criteria discussed in this paper. In Appendix E we present the R-code that can be used for such model comparisons in the *ClusterVAR* R package.

#### Sample size requirements

Our simulation showed that the LCVAR model could be estimated acceptably for 50 persons, each measured with 50 effective time points. However, estimation improved considerably for a higher number of persons and/or time points, where a higher number of persons mitigated the effects for a lower number of time points and vice versa. The number of persons necessary to accurately estimate cluster-specific parameters depended on the number of clusters. Future research could further investigate how cluster size is related to the accuracy of cluster-specific estimates. Generally, the higher the variation within-clusters (i.e., between persons of the same cluster) relative to the differences between clusters, the more time points or persons were required for accurate model estimation. In the future, more needs to be known about the compensatory relationship between the number of time points, the number of persons, and the variation within-clusters.

Our simulations did not include conditions with fewer than 50 effective time points per individual. Consequently, we are unable to assess the performance of the LCVAR model in designs with substantially fewer observations, such as typical daily diary studies. Overall, the LCVAR model can accurately capture the underlying data structure only when cluster memberships are reliably recovered $\textemdash$   an outcome that necessitates a sufficient number of time points per person. The minimum number of required time points per person will depend on factors such as the complexity of the underlying VAR model (e.g., the number of included variables) and the degree of heterogeneity across clusters and persons. Our simulation results suggest that in empirical scenarios characterized by very high within-cluster variance, more than 50 effective time points may be necessary to reliably recover cluster memberships.

### Suggestions for future research

#### Modeling within-cluster heterogeneity

We discussed several undesirable consequences of LCVAR not accommodating within-cluster variation in any of the VAR parameters (i.e., the mean, autoregressions, and cross-lagged regressions). Because LCVAR does not accommodate within-cluster variation in the means, these differences remain unaccounted in the LCVAR model. Consequently, when large within-cluster variation exists in the means in empirical data, this can lead to a biased estimation of autoregressions and cross-lagged regressions, and to correlations between residuals of the same person (see Hamaker et al., 2015). This, in turn, can lead to an over-estimation of the lag order in such cases. Thus, for data that exhibits very pronounced within-cluster differences in the means, it might be advantageous to within-person center the data before analysis with LCVAR to remove all between-person differences in means and hence all differences in means *within* clusters. However, this will also remove all such differences *between* clusters.

Another possibility to address these issues caused by within-cluster variability is through a recent extension of LCVAR which includes multilevel models within the clusters to account for within-cluster variation in person’s parameters (i.e., the mixture multilevel VAR, Ernst et al., 2024). The mixture multilevel VAR, however, has a large number of parameters and is consequently prone to estimation problems even when the number of modeled variables is low (Ernst et al., 2024). The LCVAR model on the other hand is faster and easier to estimate because it relies on fewer parameters. Additionally, LCVAR is available as a free R-implementation while the mixture multilevel VAR can so far only be estimated in Mplus using a work-around (Ernst et al., 2024; Muthén & Muthén, 1998-2017), though special cases (with few parameters) of the multilevel VAR can be estimated with the R package *dynr* (Liu et al., 2021; Ou et al., 2019). Improving estimation methods of LCVAR extensions that account for within-cluster variation could allow researchers to evaluate to what extent between-person differences are present within clusters in comparison to between clusters. This could be useful in the model evaluation and model selection of clustering models.

#### Standard errors for cluster-specific parameter estimates

The *ClusterVAR* R package currently does not provide standard errors for cluster-specific parameter estimates, such an extension could be useful to determine whether certain parameters differ significantly across clusters in the population of interest. Because LCVAR is a parametric statistical model, estimates of parameter standard errors (and thereby confidence intervals) could be estimated using derivatives of the expected value of the complete data log likelihood under the posterior distribution of cluster membership (McLachlan & Peel, 2004; Michael & Melnykov, 2016). Alternatively, a bootstrap procedure could be used to determine these standard errors conditional on the selected number of clusters and the estimated LCVAR model parameters by generating new random samples and re-estimating the model (De Menezes, 1999).

#### Selecting the best model for heterogeneous data

Researchers analyzing psychological time-series data with VAR models have a number of options for modeling heterogeneity across persons. This raises the question of which model is most appropriate for a given dataset. For instance, it is hard to judge a priori whether the heterogeneity present in the dynamics of different people is best described by a single continuous distribution using a multilevel VAR model (Bringmann et al., 2013; Driver & Voelkle, 2018; Epskamp et al., 2018; Li et al., 2022; McNeish & Hamaker, 2020; Rovine & Walls, 2006) or by distinct subgroups using an LCVAR model.

In our tutorial, we were unsure whether the true population heterogeneity contained in our data is best described by a number of classes with some variation within classes (essentially a mixture multilevel VAR; Ernst et al., 2024), or with a single-dimensional distribution such as the multivariate Gaussian distribution in a standard multilevel VAR model (e.g., McNeish & Hamaker^2020^). Both approaches can be useful descriptions of the heterogeneity between persons, and developing procedures for formal model selection would be useful.

In addition to finding the best model to capture between-person heterogeneity, researchers might be interested in comparing various models to find the one that best describes within-person changes over time. The LCVAR model cannot accommodate changes in the autoregressions or cross-lagged regressions over time, nor can it accommodate switches in cluster membership over time. However, time-varying VAR (Bringmann et al., 2017), moderated VAR (Bringmann et al., 2024; Ernst et al., 2025), or regime-switching models (Albers & Bringmann, 2020; Griffin & Li, 2016; Hamaker et al., 2009; Hamilton, 1989) can accommodate such changes over time. Future research could investigate under which conditions certain models are expected to perform better than others. Additionally, comprehensive model evaluation tools to compare these alternative models could be useful to establish which model fits a given data set best, for instance, through cross-validation (Bulteel et al., 2018; Lafit et al., 2022).

#### Extensions to the time-series model

In this paper, we restricted the underlying time-series model to a VAR with one or two lags. Although we included a linear trend in the tutorial example, researchers should adapt or extend the time-series specification to suit their data. For example, by adding a time-of-day trend (Ernst et al., 2020) or by incorporating moderators for the within-person mean (Bringmann et al., 2024). Looking ahead, it would also be valuable to extend the LCVAR framework to accommodate more general time-series models, for instance by incorporating moving average components or distributed lags (Haqiqatkhah & Hamaker, 2024; Lütkepohl, 2005).

### Conclusion

In this paper, we aimed to provide psychological researchers with everything they need to be able to use LCVAR models to analyze psychological time-series. LCVAR models are powerful models that can capture theoretically interesting between-person differences in the temporal dynamics. We hope that increased use of LCVAR models will help research better understand how persons differ in their within-person dynamics.

## Figures and Tables

**Fig. 10:**
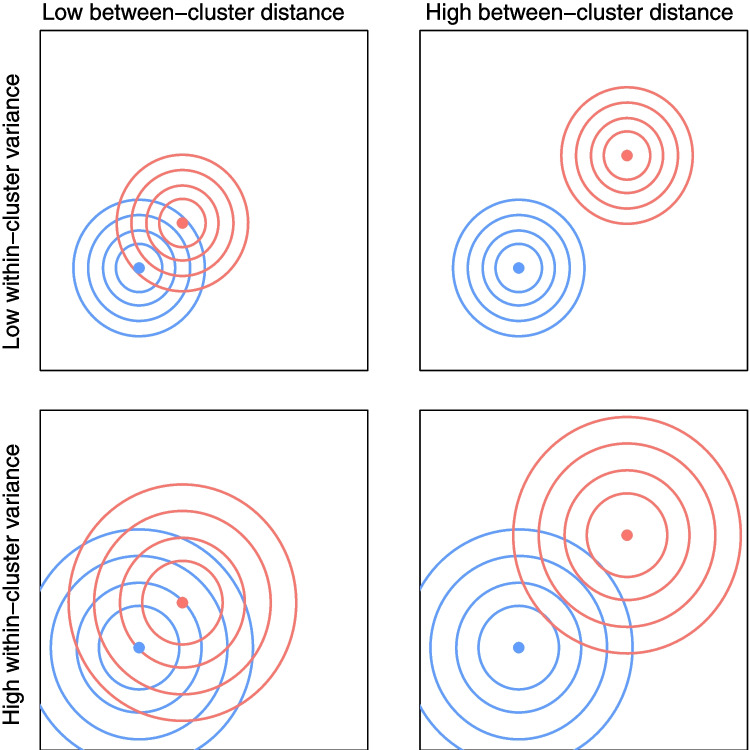
The overlap between two clusters expressed through between-cluster distances (*columns*) or through within-cluster variance (*rows*)

**Fig. 11:**
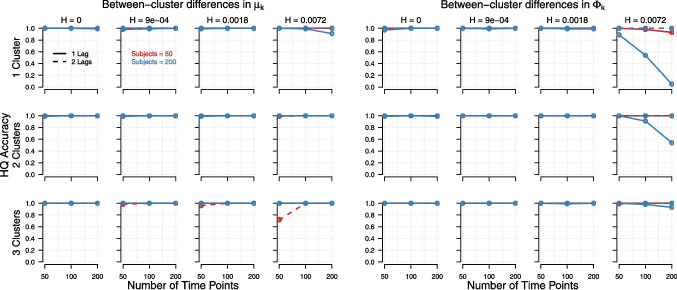
Accuracy of HQ (i.e., proportion of times HQ selected the correct lag order combination), as a function of the number of true clusters (*rows*), the within-cluster variance (*columns*), the number of time points (*x-axis*), the number of subjects (*red vs. blue*), and the number of lags (1 lag = *solid*, 2 lags = *dashed*). *Left panel*: When between-cluster differences were present in the means, $\boldsymbol{\mu }_k$. *Right panel*: The corresponding results when between-cluster differences were present in lagged effects matrix, $\boldsymbol{\Phi }_k$

**Fig. 12:**
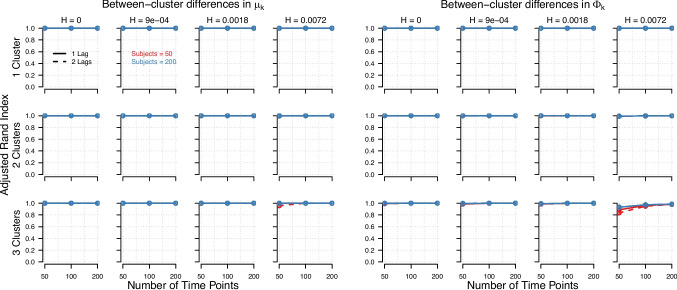
Adjusted Rand Index (ARI) values indicating cluster membership recovery, as a function of the number of true clusters (*rows*), the within-cluster variance (*columns*), the number of time points (*x-axis*), the number of subjects (*red vs. blue*), and the number of lags (1 lag = *solid*, 2 lags = *dashed*). *Left panel*: When between-cluster differences were present in the means, $\boldsymbol{\mu }_k$. *Right panel*: The corresponding results when between-cluster differences were present in the lagged effects matrix, $\boldsymbol{\Phi }_k$

**Fig. 13:**
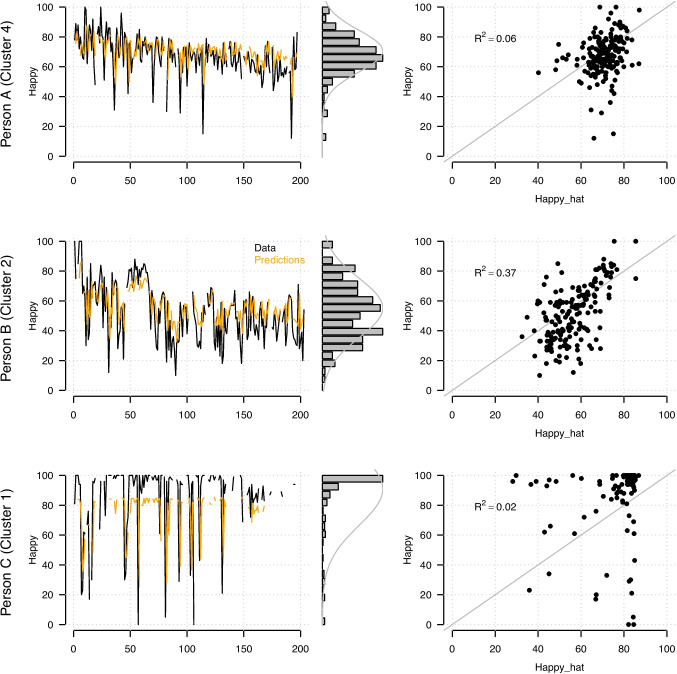
*Left*: Line plots of the variable Happy across all time points of the study (*black*) and the predictions of those time points by the LCVAR model (*orange*). The histogram shows the marginal of the empirical data. *Right*: Scatter plot showing the relationship between the predicted values, $\hat{\text {Happy}_{t}}$, and the observed values, $\text {Happy}_{t}$. The data is from the same three persons as in Fig. 5 in the main text

**Fig. 14:**
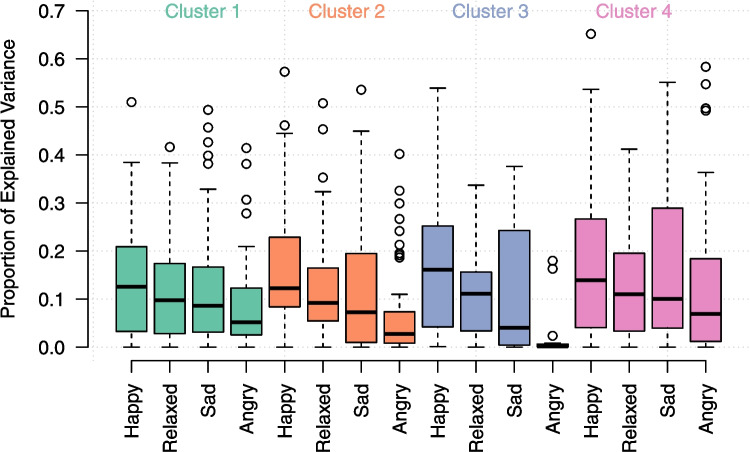
Boxplots summarizing the distributions over the proportions of explained variance ($R^2$) of persons for the four modeled variables, separately for the cluster the person was assigned to

## Data Availability

The data is available from Grommisch et al. (2020) and also in the reproducibility archive of the tutorial https://github.com/jmbh/LCVARTutorial.

## Appendix A Comparing LCVAR to Continuous Latent Variable Approaches

In popular continuous latent variable approaches, like multilevel VAR (Bringmann et al., 2013; Driver & Voelkle, 2018; Epskamp et al., 2018; Li et al., 2022; McNeish & Hamaker, 2020; Rovine & Walls, 2006) or DSEM (Hamaker et al., 2018), an individual coefficient is estimated for each person as a weighted average of that person’s own estimate and the overall average estimate (i.e., across all individuals). Thus the coefficient is pulled (shrunken) towards the overall average (De Leeuw & Meijer, 2008), which usually hinges on assumptions about the dispersion of the coefficients (e.g., a Normal distribution).

In contrast, the LCVAR model does not estimate individual coefficients, and therefore, no shrinkage occurs. Instead, cluster-specific estimates are computed for each parameter as a weighted average, where each person’s contribution is proportional to their estimated probability of belonging to the respective cluster ($\hat{\pi }_{i;k}$) and their number of effective time points.^12^ Cluster-specific estimates can be interpreted as the average parameter estimates for individuals within that cluster.

The advantage of LCVAR is that it can account for structural and qualitative differences between clusters (e.g., a lag 1 model for Cluster 1 and a lag 2 model for Cluster 2), allowing for between-cluster differences of nearly any form. The drawback is that, while continuous approaches account for individual deviations from the average, the LCVAR model does not capture any individual deviation from the cluster-specific estimate $\textemdash$ that is, it does not account for within-cluster variance.

## Appendix B Formulas for information criteria

The best-fitting combination of lag orders for a given number of clusters can be determined through the following time-series information criteria which weigh forecasting precision against the number of effective time points and the number of time-series parameters: HQ and SC (Akaike, 1969, 1971, 1974; Quinn, 1980).

8 $$\begin{aligned} \text {HQ} = \sum _{k = 1}^{K} \tau _k \left( \text {log}\left( \text {det}(\boldsymbol{\hat{\Sigma }}_k) \right) + \frac{2p_k(m^2) \text {log}\left( \text {log}\left( \sum _{i = 1}^{N} \hat{\pi }_{i\, k} T_{i\; k}\right) \right) }{\sum _{i = 1}^{N} \hat{\pi }_{i\, k} T_{i\; k}}\right) . \end{aligned}$$

9 $$\begin{aligned} \text {SC} = \sum _{k = 1}^{K} \tau _k \left( \text {log}\left( \det (\hat{\boldsymbol{\Sigma }}_k) \right) + \frac{2p_k(m^2) \text {log}\left( \sum _{i = 1}^{N} \hat{\pi }_{i\, k} T_{i\; k}\right) }{\sum _{i = 1}^{N} \hat{\pi }_{i\, k} T_{i\; k}}\right) . \end{aligned}$$

In the equations above, *m* denotes the number of endogenous variables in the VAR equation and $p_k$ denotes the lag order in a given cluster, *k*.

The following information criteria can determine the best-fitting number of clusters, given a lag order that will be the same for each cluster: BIC (Schwarz, 1978) and ICL (Biernacki et al., 2000).

10 $$\begin{aligned} \text {BIC} = -2 \times \text {log}[\mathcal {L}(\boldsymbol{ \Theta }; \boldsymbol{Y})] + P \times \text {log}\left( \sum _{k = 1}^{K} \sum _{i = 1}^{N} \hat{\pi }_{i\, k} T_{i\; k}\right) . \end{aligned}$$

11 $$\begin{aligned} \text {ICL} = \text {BIC} + \text {log}\left( \sum _{k = 1}^{K} \sum _{i = 1}^{N} \hat{z}_{i\, k} \hat{\pi }_{i\, k} \right) . \end{aligned}$$

In the equations above, $\text {log}[\mathcal {L}(\boldsymbol{ \Theta }; \boldsymbol{Y})]$ denotes the log likelihood value of a given LCVAR model, *P* denotes all parameters in that LCVAR model, and $\hat{z}_{i\, k}$ is an indicator variable that is 1 if person *i* is assigned into cluster *k* and is 0 otherwise (where persons are assigned into a cluster based on their modal cluster membership probabilities, $\hat{\pi }_{i\, k}$).

## Appendix C Overlap between clusters

As illustrated in Fig. 10, any amount of overlap between clusters can be achieved by manipulating either the between-cluster distances between cluster-specific parameters, or by manipulating the within-cluster variance around these parameters.

Regarding simulation factor (5) whether *between-cluster differences* were contained only in $\boldsymbol{\Phi }_k$ or only in $\boldsymbol{\mu }_k$. In the following we provide the details on how we implemented between-cluster differences in our simulation. When between-cluster differences were in $\boldsymbol{\mu }_k$, we manipulated four cluster-specific parameters to differ across any two clusters; when differences were in $\boldsymbol{\Phi }_k$, we manipulated 10 cluster-specific parameters to differ across any two clusters. We deem this a valid set-up because $\boldsymbol{\mu }_k$ contains less parameters overall than $\boldsymbol{\Phi }_k$ and thus in practice between-cluster differences in $\boldsymbol{\mu }_k$ are likely occurring on a smaller number of parameters than differences in $\boldsymbol{\Phi }_k$.

To prevent that our results for the overlap between clusters are always driven by the two clusters with the most/least overlap, we ensure that for any two clusters the between-cluster distance is the exact same as the distance between any other two clusters. (1) If the between-cluster distances were contained in $\boldsymbol{\Phi }_k$, then across the different clusters in a generated data set, any two $\boldsymbol{\Phi }_k$ differed in ten parameters (in two positions in autoregressive parameters and in eight positions in cross-lagged regression parameters) by 0.3 while all other parameters were equal across these two $\boldsymbol{\Phi }_k$.

To keep the between-cluster differences equal between the lag 1 and the lag 2 conditions, these between-cluster distances were always contained in only the lag 1 parameters, even in conditions in which lag 2 parameters were present. All lag 1 parameters contained in the $\boldsymbol{\Phi }_k$ were equal to either $-0.3, 0, 0.2, 0.3,$ or 0.5. In the lag 2 conditions, all lag 1 parameters were determined as specified here while all lag 2 cross-lagged regressions were set to zero and all lag 2 autoregressive effects were set to 0.2. (2) If the between-cluster distances were contained in the means, $\boldsymbol{\mu }_k$, the $\boldsymbol{\mu }_k$s of different clusters differ from each other in four positions by 0.75 for any two clusters. All means contained in $\boldsymbol{\mu }_k$ were equal to either $-0.75, 0$, or 0.75. Thus, the absolute sum of between-cluster distances in cluster-average parameters is 3 in both, the conditions where between-differences were contained in $\boldsymbol{\Phi }_k$ and the conditions where between-differences were contained in $\boldsymbol{\mu }_k$.

## Appendix D Additional simulation results

Here we present additional tables and figures to supplement the Simulation Results section of the manuscript. Regarding model recovery, the main effects (i.e., averaged over all other simulation factors) for each of the six simulation factors on model recovery are summarized in Table 2. The proportion of times the HQ selected the correct combination of lag orders across simulation conditions is presented in Fig. 11. The average over- and under-estimation of the number of clusters by the ICL is shown in Table 3 for each of the six simulation factors.

Regarding the estimation recovery of parameters, the recovery of cluster memberships, measured through the ARI, across simulation conditions is shown in Fig. 12. The main effects (i.e., averaged over all other simulation factors) of the six simulation factors on estimation recovery of $\boldsymbol{\mu }_k$, $\boldsymbol{\Phi }_k$, and cluster-memberships are summarized in Table 4.

**Table 2:** Main effects (i.e., averaged over all other simulation factors) for model recovery per simulation factor. The table shows the proportion of correctly identified models for each simulation factor

		SC (lags)	HQ (lags)	BIC (clusters)	ICL (clusters)
Between-cluster differences	$\boldsymbol{\mu }_k$	0.8988	0.9963	0.7853	0.7855
	$\Phi _k$	0.9212	0.9837	0.8462	0.8462
Number of lags	1 lag	1.0000	0.9825	0.8788	0.8788
	2 lag	0.8199	0.9975	0.7528	0.7529
Number of clusters	1 Cluster	0.9947	0.9810	0.8946	0.8946
	2 Clusters	0.9051	0.9936	0.8651	0.8652
	3 Clusters	0.8301	0.9953	0.6877	0.6878
$N_{subj}$	50 Persons	0.8256	0.9969	0.7676	0.7676
	200 Persons	0.9943	0.9831	0.8640	0.8641
$N_{T}$	50 Time points	0.8224	0.9941	0.7478	0.7478
	100 Time points	0.9080	0.9933	0.8489	0.8489
	200 Time points	0.9995	0.9826	0.8507	0.8509
Within-cluster variance	0	0.9108	0.9990	0.8819	0.8819
	9e-04	0.9132	0.9990	0.8815	0.8815
	0.0018	0.9137	0.9988	0.8792	0.8792
	0.0072	0.9021	0.9632	0.6206	0.6208

**Table 3:** Proportion of times the ICL over-estimates or under-estimates the number of clusters (main effects per simulation factor)

		ICL over-estimation	ICL under-estimation
Between-cluster differences	$\boldsymbol{\mu }_k$	0.0437	0.1708
	$\Phi _k$	0.0831	0.0707
Number of lags	1 lag	0.0692	0.0520
	2 lag	0.0576	0.1894
Number of clusters	1 Cluster	0.1054	0.0000
	2 Clusters	0.0506	0.0842
	3 Clusters	0.0342	0.2780
$N_{subj}$	50 Persons	0.0192	0.2131
	200 Persons	0.1076	0.0283
$N_{T}$	50 Time points	0.0069	0.2453
	100 Time points	0.0451	0.1060
	200 Time points	0.1382	0.0108
Within-cluster variance	0	0.0000	0.1181
	9e-04	0.0000	0.1185
	0.0018	0.0000	0.1208
	0.0072	0.2536	0.1256

**Table 4:** Main effects (i.e., averaged over all other simulation factors) for estimation recovery per simulation factor. The table shows either the mean absolute deviation between the parameter estimate and the parameter (here low values indicate good recovery) or the adjusted Rand index (here high values indicate good recovery)

		MAD for $\boldsymbol{\mu }_k$	MAD for $\boldsymbol{\Phi }_k$	ARI
Between-cluster differences	$\boldsymbol{\mu }_k$	0.0196	0.0171	0.9996
	$\Phi _k$	0.0327	0.0180	0.9945
Number of lags	1 lag	0.0194	0.0169	0.9974
	2 lag	0.0329	0.0182	0.9966
Number of clusters	1 Cluster	0.0206	0.0136	1.0000
	2 Clusters	0.0244	0.0177	0.9993
	3 Clusters	0.0335	0.0213	0.9917
$N_{subj}$	50 Persons	0.0348	0.0226	0.9961
	200 Persons	0.0175	0.0126	0.9980
$N_{T}$	50 Time points	0.0347	0.0227	0.9936
	100 Time points	0.0253	0.0169	0.9983
	200 Time points	0.0185	0.0131	0.9992
Within-cluster variance	0	0.0226	0.0153	0.9998
	9e-04	0.0232	0.0157	0.9995
	0.0018	0.0240	0.0163	0.9992
	0.0072	0.0348	0.0230	0.9897

Note. MAD stands for mean absolute deviation, ARI stands for adjusted Rand index

**Table 5:** Main effects (i.e., averaged over all other simulation factors) for estimation recovery per simulation factor. The table shows mean absolute deviation between the parameter estimate and the parameter (low values indicate good recovery) for autoregressions and cross-regressions at lag 1 (i.e., the first *m* columns of $\boldsymbol{\Phi }_k$)

		MAD for AR	MAD for CR
Between-cluster differences	$\boldsymbol{\mu }_k$	0.0198	0.0168
	$\Phi _k$	0.0267	0.0168
Number of lags	1 lag	0.0207	0.0161
	2 lag	0.0258	0.0175
Number of clusters	1 Cluster	0.0209	0.0124
	2 Clusters	0.0230	0.0170
	3 Clusters	0.0259	0.0209
$N_{subj}$	50 Persons	0.0272	0.0222
	200 Persons	0.0194	0.0114
$N_{T}$	50 Time points	0.0276	0.0221
	100 Time points	0.0226	0.0161
	200 Time points	0.0195	0.0122
Within-cluster variance	0	0.0153	0.0153
	9e-04	0.0164	0.0157
	0.0018	0.0185	0.0162
	0.0072	0.0429	0.0199

Note. MAD stands for mean absolute deviation, AR for autoregressions, CR for cross-lagged regressions

## Appendix E ClusterVAR code for selection number of clusters and lag order combination

In the manuscript, we mentioned that in case researchers want to select the lag order in addition to the number of clusters, we recommend that they first select the number of clusters while fixing the lag order to 1 across all clusters. Then, in a subsequent step, they can fix the resulting number of clusters while selecting the lag-order combination across clusters using one of the time-series information criteria discussed in this paper. In the *ClusterVAR* R package this can be achieved by first estimating all possible lag order combinations between one and two lags for each number of clusters between 1 and a given number *K*: $\texttt {output <- LCVAR(..., Clusters \!=\! 1:K,}$ $\texttt {Lags} \texttt { = 1:2)}$. Then, models with different number of clusters where the lag order in each cluster is fixed to 1 can be compared through: summary(object = output, show = "GNL", Number_of_Lags = 1). Subsequently, all lag order combinations for models with the number of clusters equal to a given number, *k*, can be compared through: summary(object = output, show = "GNC", Number_of_Clusters = k).

## Appendix F Tutorial: Residual analysis

There are many useful ways to visually assess absolute model fit. Here we choose to plot the observed time-series (black lines) together with the (one time step forward-) predicted time-series (orange line; left panel of Fig. 13) and plot the predicted values (*x*-axis) against the observed values (*y*-axis; right panel). One could also plot the residuals over time or inspect the distribution of residuals using a histogram. Here, we only use the two mentioned visualizations to inspect absolute model fit for the variable Happy for the same three persons that we already showed in Fig. 5 in the main text.

For Person A, we see that the observed data and the predictions align relatively well at the beginning of the time series, but then the predictions seem to diverge from the observed data. This is because we observed a clear negative trend in this time series (see Fig. 5). However, Person A was estimated to be in Cluster 4, which has a weak positive linear trend (see Fig. 7). This explains why the predictions become worse over time. Plotting the predictions of the variable Happy (*x*-axis) against the corresponding observed values (*y*-axis) shows that there is a weak positive relationship ($R^2$=0.06) between predictions and observed data.

For Person B, we find a much better fit, which can be seen in the time-series plots where we see that the predictions are roughly aligned in with the observed data across the entire time series. We also see in the right panel that there is a stronger linear relationship ($R^2$=0.37) between the predictions of Happy and the corresponding observed values compared to Person A.

Finally, for Person C, we observe severe model misfit. As discussed in the main text, the values for Happiness seem to switch between values close to 100 and values throughout the rest of the scale. The cluster-specific VAR model in Cluster 1 has a mean around 60, which pulls predictions towards this value: the maximum values at around 100 are underestimated, while the deviations closer towards the lower end of the scale are overestimated. The poor correspondence between predictions and observed data is also clear from the scatter plot between predictions of Happy and corresponding observed values in the right panel. We see that observed values at 100 are systematically underestimated by the predictions around 80. Beyond that, we also see that some values are severely overestimated while others are severely underestimated.

Note that in the above analysis of absolute misfit, we focused on systematic misfit. However, one can also have low absolute model fit in terms of prediction errors (such as $R^2$) when the model fits perfectly. For example, one could generate data from a VAR model with very small lagged effects parameters and relatively large innovation variances. While a fitted VAR model (or the true VAR model for that matter) would lead to poor predictions in terms or prediction errors, we still would be capturing all the structure that is in the data.

Inspecting model fit is important because the conclusions drawn from the estimated LCVAR model likely depend on how well the model actually fits the data and how much model fit differs between people. If the model fits extremely well, and for everyone, one can conclude that the LCVAR model captured most of the structure in the data for most persons. However, if model fit is poor (for some persons), this could provide insights for improvements of the model (e.g., adding a trend) or at least should lead to less strong conclusions.

Next to inspecting model fit for every person, we can also compute summary statistics on prediction errors. For example, we could compute the distribution of the proportion of explained variance ($R^2$) for each variable, separately for persons in the different clusters, as shown in Fig. 14. We see that the $R^2$ are generally below 0.2, which means that the large majority of the variance remains unexplained for most variables and persons, in all clusters. We see that negatively valenced emotions tend to have lower $R^2$, which likely has to do with the fact that those are often scored at the low end of the measurement scale. This leads to (highly) skewed distributions, which the VAR model cannot approximate well. This is especially extreme for the variable Angry in Cluster 3. For persons in this cluster, Angry is scored almost entirely at 0 or very close to it, leading to very small standard deviations. This is consistent with Fig. 7, which shows that Sad and Angry have means very close to 0 in this Cluster. It can also be seen from time-series plots of each individual ordered by cluster membership, which can be found in the reproducibility archive. In such cases, the VAR model will lead to very poor predictions, as can be seen by the $R^2$ close to zero for Angry in Cluster 3 in Fig. 14.

It is important to keep in mind that a VAR model can make predictions that show a large variety of trends and marginal distributions, such as for Person C in Fig. 13. This is because the predictor variables exhibit these trends and distributions, and the predictions of the VAR models are linear combinations of those predictor variables. Importantly, this does not mean that the VAR model as a generative model can produce such trends and distributions. For example, we could take the VAR model from Cluster 1 to which Person C belongs, initialize it with the values of Person C at $t=0$ and run the model for 200 time points. This time series would look nothing like the empirical data or the predictions of Person C. Instead, we would see a slightly autocorrelated stationary process with a Gaussian marginal distribution (see also Appendix G in Haslbeck et al., 2023, for a demonstration).

## Footnotes

1 For clustering models where persons switch between different clusters over time, see Cabrieto et al. (2018), Ou et al. (2019), or Hamaker et al. (2016).

2 For initialization methods, see Ernst et al. (2020).

3 https://osf.io/92mj6

4 $\boldsymbol{\Phi }_i$ contains 36 parameters in the lag 1 case and 72 parameters in the lag 2 case.

5 If the parameters in a generated $\boldsymbol{\Phi }_i$ implied a non-stationary process, the parameters were re-sampled. We verified after the data generation that the within-cluster variance of the generated data was as specified, despite the practice of re-sampling certain $\boldsymbol{\Phi }_i$.

6 Except for the three-cluster condition, where the proportions were 0.34, 0.34, and 0.32.

7 The number of candidate models with different lag 1 and lag 2 combinations thus varied across the different true underlying number of clusters. For example, if the true number of clusters was 1, only two different lag order combinations were compared: (A) a lag 1 model for this cluster, or (B) a lag 2 model for this cluster. If the number of underlying clusters was 3, however, four different lag order combinations were compared: (A) a lag 1 model for all clusters, (B) two lag 1 models and one lag 2 model, (C) one lag 1 model and two lag 2 models, or (D) three lag 2 models.

8 We employed version 0.0.5 in this simulation. Subsequent updates to the package do not impact the results presented in this study.

9 Because in our simulation study there were no missing data, the number of effective time points and the number of observed time points are nearly identical. For an explanation of the distinction between number of observed time points and number of effective time points in empirical data, see Section “Lag orders and the effective number of time points”.

10 For a discussion on the distinction between these different models, see Appendix A.

11 This approach aligns with the view that empirical data typically lack a true number of clusters; instead, clustering provides a meaningful summary of the underlying structure.

## Declarations

### Code availability

The code to reproduce the simulation study and all results and figures shown in the simulation section can be found at https://osf.io/92mj6; the empirical data and the code to reproduce the tutorial can be found at https://github.com/jmbh/LCVARTutorial.
